# Supplementary material for: Virome heterogeneity and connectivity in waterfowl and shorebird communities
Source: ISME J. 2019 Jun 25;13(10):2603–16. doi: 10.1038/s41396-019-0458-0 (PMC6775988; doi:10.1038/s41396-019-0458-0)
Supplement: Supplementary file 1 — Supplemental Meterials [file 41396_2019_458_MOESM1_ESM.pdf]

Virome heterogeneity and connectivity in waterfowl and shorebird communities

Michelle Wille, Mang Shi, Marcel Klaassen, Aeron C. Hurt, Edward C. Holmes

Contents

Supplemental Methods ... 3

Table S1. Metadata for each library in this study ... 6

Table S2. Sequence alignment lengths for phylogenetic analysis ... 7

Table S3. Viruses (>1000bp) identified in this study ... 8

Figure S1. Correlation of laboratory and sequencing metrics ... 10

Figure S2. Schematic overview of novel viruses revealed in this study ... 11

Figure S3. Phylogenetic tree of the ORF1ab, including the RdRp, of Avastroviruses ... 12

Figure S4. Partial RdRp phylogeny of members of the Avastroviruses ... 13

Figure S5. Phylogeny of the polyprotein, containing the RdRp, of select members of the *Picornaviridae* ... 14

Figure S6. Phylogeny of the polyprotein, containing the RdRp, of select members of the Megriviruses ... 15

Figure S7. Phylogenetic tree of the VP1, containing the RdRp, of the rotaviruses ... 16

Figure S8. Phylogenetic tree of the P protein of avian hepatitis B viruses ... 17

Figure S9. Phylogenetic tree of the ORF1ab, containing the RdRp, of the *Coronaviridae* ... 18

Figure S10. Phylogeny of the partial RdRp of gammacoronaviruses ... 19

Figure S11. Phylogeny of the partial RdRp of deltacoronaviruses ... 20

Figure S12. Phylogenies of the HA genes of H9 and H3 influenza A viruses containing all wild bird sequences ... 21

Figure S13. Phylogenies of the NA and NP genes of influenza A viruses, containing all wild bird sequences ... 22

Figure S14. Phylogeny of the F gene of avian avulavirus Type 1 Class II Genotype 1 ... 23

Figure S15. Abundance of avian viral families identified in each library ... 24

Figure S16. Alpha diversity of avian viral families identified in each library ... 25

Figure S17. Alpha diversity of avian viral genera identified in each library ... 26

Figure S18. Alpha diversity of avian viral species characterised in this study ... 27

Figure S19. Non-metric multidimensional scaling (NMDS) plot applying the bray curtis dissimilarity matrix for viral abundance and diversity ... 28

Figure S20. Effect of IAV on Alpha Diversity of Anseriiform viromes ... 29

Figure S21. Effect of coronavirus on alpha diversity of Anseriiform viromes ... 30

Figure S22. Differential abundance of viral families in libraries from Anseriiformes and Charadriiformes 31

Figure S23. Differential abundance of viral genera in the family *Picornaviridae* in libraries from Anseriiformes and Charadriiformes 32

Figure S24. Differential abundance (log 2 fold change) of viruses 33

Figure S25. Co-phylogeny demonstrating a discordance between host phylogenetic relationships and virome composition 34

Figure S26. Co-phylogeny demonstrating the discordance between host phylogenetic relationships and virome composition 35

Supplemental Methods

Sample selection

Samples were collected as part of a long-term IAV surveillance study (1, 2). A combination or oropharyngeal and cloacal samples were collected using a sterile-tipped swab and were placed in viral transport media (VTM, Brain-heart infusion broth containing 2x106 122 U/l penicillin, 0.2 mg/ml streptomycin, 0.5 mg/ml gentamicin, 500 U/ml amphotericin B, Sigma). Samples were kept cool immediately following collection (4-12C), and subsequently placed at -80C for long-term storage.

RNA virus discovery

We selected 10-15 samples per species for RNA extraction. RNA was extracted and libraries constructed as per (3). Briefly, RNA was extracted from swab samples using the MagMax mirVana™ Total RNA Isolation Kit (Thermo Fisher Scientific) on the KingFisher™ Flex Purification System (Thermo Fisher Scientific). Ten samples with the highest concentration were pooled (based upon species) using equal concentrations using the RNeasy MinElute Cleanup Kit (Qiagen). Libraries were constructed using the TruSeq total RNA library preparation protocol (Illumina), and host rRNA was removed using the Ribo-Zero Gold Kit (Illumina). Paired end sequencing (100bp) of the RNA library was performed on an Illumina HiSeq 2500 platform at the Australian Genome Research Facility (AGRF, Melbourne).

We used the bioinformatics pipeline reported in (3-5). Accordingly, sequence reads were demultiplexed and trimmed with Trimmomatic, and contigs were *de novo* assembled using Trinity (6). No filtering of host/bacterial reads was performed before assembly. All assembled contigs were compared to the entire nonredundant nucleotide (nt) and protein (nr) database using blastn and diamond blast (7), respectively, setting an e-value threshold of 1×10^{-10} to remove potential false positives. Abundance estimates for all contigs were determined using the RSEM algorithm implemented in Trinity.

RNA sequencing of rRNA depleted libraries resulted in an average of 50,906,440 reads (range 44,178,936-64,149,180) assembled into a mean of 27,108 contigs (range 22,175-496,319) (Table S1). There was no significant correlation between RNA concentration and the number of reads, contigs or abundance demonstrating such that this was not a source of bias (Fig S1).

Virus genome annotation and phylogenetic analysis

Contigs were annotated, and phylogenetic trees inferred as per (3). Briefly, contigs greater than 1000bp in length were inspected using Geneious R10 (Biomatters, New Zealand), and open reading frames corresponding to predicted genome architectures based on the closest reference genomes were interrogated. Reads were subsequently mapped back to viral contigs to identify mis-assembly using bowtie2 (8). Viruses with full length genomes, or incomplete genomes that possess the full-length RNA-dependant RNA polymerase (RdRp) gene, were used for phylogenetic analysis. Sequences of the polyprotein or gene encoding for the RdRp were aligned using MAFFT (9), and gaps and ambiguously aligned regions were stripped using trimAL (10). Final alignment lengths for each data set are presented in Table S2. The most appropriate amino acid substitution model was then determined for each data set, and maximum likelihood trees were estimated using PhyML 3.0 (11) with 1000 bootstrap replicates. Novel viral species were identified as those that had <90% RdRp protein identity, or <80% genome identity to previously described viruses.

Diversity and abundance across libraries

We used diversity metrics commonly used in microbial community ecology to understand composition and the factors that may drive variation in the composition of avian viromes. The alpha-diversity and beta-diversity indices are measures of within- and between-sample diversity, respectively, and have been associated with various ecological traits and biological phenotypes since the first influential human or environmental metagenomics studies (e.g. (12, 13). Although often associated with microbiome research, they have also been employed in viral ecology (e.g. (14-16), and we extend their use here.

References

1. Ferenczi M. Avian influenza virus dynamics in Australian wild birds. PhD Thesis: Deakin University; 2016.
2. Ferenczi M, Beckmann C, Warner S, Loyn R, O'Riley K, Wang X, et al. Avian influenza infection dynamics under variable climatic conditions, viral prevalence is rainfall driven in waterfowl from temperate, south-east Australia. Vet Res. 2016;47:23.
3. Wille M, Eden JS, Shi M, Klaassen M, Hurt AC, Holmes EC. Virus-virus interactions and host ecology are associated with RNA virome structure in wild birds. Mol Ecol. 2018:doi: 10.1111/mec.14918.
4. Shi M, Lin XD, Chen X, Tian JH, Chen LJ, Li K, et al. The evolutionary history of vertebrate RNA viruses. Nature. 2018;556(7700):197-202.
5. Shi M, Lin XD, Tian JH, Chen LJ, Chen X, Li CX, et al. Redefining the invertebrate RNA virosphere. Nature. 2016;540(7634):539-43.

6. Grabherr MG, Haas BJ, Yassour M, Levin JZ, Thompson DA, Amit I, et al. Full-length transcriptome assembly from RNA-Seq data without a reference genome. Nature Biotech. 2011;29(7):644-52.
7. Buchfink B, Xie C, Huson DH. Fast and sensitive protein alignment using DIAMOND. Nat Methods. 2015;12(1):59-60.
8. Langmead B, Salzberg SL. Fast gapped-read alignment with Bowtie 2. Nat Methods. 2012;9(4):357-U54.
9. Katoh K, Standley DM. MAFFT multiple sequence alignment software version 7: improvements in performance and usability. Mol Biol Evol. 2013;30(4):772-80.
10. Capella-Gutierrez S, Silla-Martinez JM, Gabaldon T. trimAl: a tool for automated alignment trimming in large-scale phylogenetic analyses. Bioinformatics. 2009;25(15):1972-3.
11. Guindon S, Dufayard JF, Lefort V, Anisimova M, Hordijk W, Gascuel O. New algorithms and methods to estimate maximum-likelihood phylogenies: assessing the performance of PhyML 3.0. Systematic biology. 2010;59(3):307-21.
12. Huttenhower C, Gevers D, Knight R, Abubucker S, Badger JH, Chinwalla AT, et al. Structure, function and diversity of the healthy human microbiome. Nature. 2012;486(7402):207-14.
13. Fierer N, Leff JW, Adams BJ, Nielsen UN, Bates ST, Lauber CL, et al. Cross-biome metagenomic analyses of soil microbial communities and their functional attributes. Proc Natl Acad Sci U S A. 2012;109(52):21390-5.
14. McCann A, Ryan FJ, Stockdale SR, Dalmasso M, Blake T, Ryan CA, et al. Viromes of one year old infants reveal the impact of birth mode on microbiome diversity. Peerj. 2018;6.
15. Roux S, Emerson JB, Eloe-Fadrosh EA, Sullivan MB. Benchmarking viromics: an in silico evaluation of metagenome-enabled estimates of viral community composition and diversity. Peerj. 2017;5.
16. Hannigan GD, Meisel JS, Tyldsley AS, Zheng Q, Hodkinson BP, SanMiguel AJ, et al. The Human Skin Double-Stranded DNA Virome: Topographical and Temporal Diversity, Genetic Enrichment, and Dynamic Associations with the Host Microbiome. mBio. 2015;6(5).

Table S1. Metadata for each library in this study.

Host Taxonomy	Species	Scientific Name	Bird capture/collection method	Sample location, date	RNA conc (pg/ul)	No. Paired Reads	No. Contigs	Total Viral Abundance	Avian Viral Abundance
Charadriiformes, Scolopacidae	Red-necked Stint	*Calidris ruficollis*	Cannon netting	Western Treatment Plant, December 2012	10300	25,007,631	95246	5.89	8.48e-02
Charadriiformes, Scolopacidae	Curlew Sandpiper	*Calidris ferruginea*	Cannon netting	Western Treatment Plant, December 2012	10300	32,074,590	22175	0.15	1.84e-04
Charadriiformes, Scolopacidae s	Sharp-tailed Sandpiper	*Calidris acuminata*	Cannon netting	Western Treatment Plant, December 2012	4700	31,690,395	40843	0.37	8.46e-02
Charadriiformes, Charadridae	Red-capped Plover	*Charadrius ruficapillus*	Cannon netting	Western Treatment Plant, December 2012	9000	26,073,463	95577	3.20	2.301e-02
Anseriiformes, Antidae, Anatinae	Pacific Black Duck	*Anas superciliosa*	Hunter shot	SW Victoria, March/April 2017	38700	24,873,979	496319	0.47	1.98e-03
Anseriiformes, Antidae, Anatinae	Grey Teal	*Anas gracilis*	Hunter shot	SW Victoria, March/April 2017	16500	22,984,396	435173	0.97	5.078e-02
Anseriiformes, Antidae, Tadorninae	Australian Shelduck	*Tadorna tadornoides*	Hunter shot	SW Victoria, March/April 2017	14900	22,089,468	540006	0.21	3.54e-02
Anseriiformes, Antidae, UR[1]	Australian Wood Duck	*Chenonetta jubata*	Hunter shot	SW Victoria, March/April 2017	30400	22,142,942	531670	0.39	2.45e-03
Anseriiformes, Antidae, UR	Pink-eared duck	*Malacorhynchus membranaceus*	Hunter shot	SW Victoria, March/April 2017	30600	22,142,051	209969	10.67	4.72e-02

1. UR: Unresolved Subfamily. Australian Wood Ducks are occasionally placed in subfamily Anatinae or Tadorninnae. Pink-eared Ducks are occasionally placed in subfamily Tadorninae, Oxyurinae, or Dendrocheninae.

Table S2. Sequence alignment lengths for phylogenetic analysis

Group	Taxonomy level	Gene	Nucleotide or amino acid	Alignment length	Alignment length following trimAL
ssRNA	*Astroviridae*, Avastrovirus	ORF1ab (includes RdRp)	Amino acid	2029 aa	1449 aa
ssRNA	*Astroviridae*, Avastrovirus	partial ORF1ab (includes RdRp)	Nucleotide	398 bp	
ssRNA	*Coronaviridae*	ORF1ab (includes RdRp)	Amino acid	10805 aa	6824 aa
ssRNA	*Coronaviridae*, gammacoronavirus	partial ORF1ab (includes RdRp)	Nucleotide	290bp	
ssRNA	*Coronaviridae*, deltacoronavirus	partial ORF1ab (includes RdRp)	Nucleotide	290bp	
ssRNA	*Calciviridae*	Polyprotein (includes RdRp)	Amino acid	3836 aa	2401 aa
dsRNA	*Picorbirnaviridae*	Segment 2 (includes RdRp)	Amino acid	1071 aa	527 aa
ssRNA	*Picornaviridae*	Polyprotein (includes RdRp)	Amino acid	5820 aa	2546 aa
ssRNA	*Orthomyxoviridae,* Influenza A virus	HA	Nucleotide	1734 bp	
ssRNA	*Orthomyxoviridae,* Influenza A virus	NA	Nucleotide	1446 bp	
ssRNA	*Orthomyxoviridae,* Influenza A virus	NP	Nucleotide	1580 bp	
ssRNA	*Paramyxoviridae,* Avian avulavirus 1, class II	F gene	Nucleotide	1662bp	
dsRNA	*Reoviridae,* Rotavirus	VP1 (includes RdRp)	Amino acid	1198 aa	
DNA	*Hepadnaviridae, Avihepandavirus?*	DNA directed DNA polymerase P protein	Amino acid	484 aa	401 aa

Table S3. Viruses (>1000bp) identified in this study.

Host Species	Viral Family	Viral Genus	Virus Species	New species	Complete?	No. Reads Mapped	Closest Blastx hit	pident (Blastx)	e-value
Red-necked Stint	Paramyxoviridae	Avulavirus	Avian avulavirus 1	No	Yes	178000	AFU34346.1 polymerase [Avian avulavirus 1]	99	0
Red-necked Stint	Picornaviridae	Unassigned	Red-necked Stint Picornavirus B-like	Yes	Yes	9287	YP_004564600.1 polyprotein [Pigeon picornavirus B]	45.7	0
Red-necked Stint	Astroviridae	Avastrovirus	Red-necked Stint Astrovirus Group 4	Yes	Yes	6302	CAB95006.3 nonstructural protein; putative RNA-dependant RNA polymerase [Avastrovirus 1]	53	7.40E-157
Red-necked Stint	Picornaviridae	Gallivirus	Red-necked stint Gallivirus	Yes	Capsid, RdRp	6523	ADL38958.1 polyprotein [Passerivirus A1]	32.5	3.00E-141
Sharp-tailed Sandpiper	Coronaviridae	Deltacoronavirus	Deltacoronavirus	Yes	ORF1ab	43518	YP_005352837.1 orf1ab gene product [White-eye coronavirus HKU16]	73.1	0
Sharp-tailed Sandpiper	Picornaviridae	Unassigned	Sharp-tailed Sandpiper Picornavirus B-like	Yes	sort of	7269	YP_004564600.1 polyprotein [Pigeon picornavirus B]	58.7	1.10E-186
Red-capped Plover	Picornaviridae	Megrivirus	Sharp-tailed Sandpiper Megrivirus	Yes	Yes	9107	YP_009345890.1 polyprotein [Goose megrivirus]	48.3	0
Pacific Black Duck	Picornaviridae	Megrivirus	Pacific Black Duck Megrivirus	Yes	sort of	671	YP_009030047.1 polyprotein [Duck megrivirus]	84.2	1.00E-271
Grey Teal	Coronaviridae	Gammacoronavirus	Gammacoronavirus	No	no	8089	AKF17723.1 ORF1a [Duck coronavirus]	79.5	0
Grey Teal	Caliciviridae	Unassigned	Grey Teal Calicivirus	Yes	Yes	2082	AFH89833.1 polyprotein [Turkey calicivirus]	42	0
Grey Teal	Orthomyxoviridae	Influenza A	Influenza A	H9N1	Yes	1416	ACZ15987.1 hemagglutinin [Influenza A virus (A/mallard/Switzerland/WV1070805/2007(H9N2))]	98.2	0
Grey Teal	Picobirnaviridae	Picobirnavirus	Grey Teal Picobirnavirus A	Yes	Segment 1 only	340	AGK45547.1 hypothetical protein; partial [Fox picobirnavirus]	37.2	1.70E-64
Grey Teal	Picobirnaviridae	Picobirnavirus	Grey Teal Picobirnavirus B	Yes	2 segments, both partial	420	ALL29321.1 RNA-dependent RNA polymerase [Human picobirnavirus]	64.7	7.40E-195
Grey Teal	Picobirnaviridae	Picobirnavirus	Grey Teal Picobirnavirus C	Yes	2 segments, both partial	164	AMP18960.1 RNA-dependent RNA polymerase [Otarine picobirnavirus]	74.6	2.60E-127
Grey Teal	Reoviridae	Rotavirus	Grey Teal Rotavirus F-like	Yes	partial VP1	858	AIW53371.1 VP1; partial [Rotavirus F]	70.4	8.20E-28
Grey Teal	Reoviridae	Rotavirus	Duck Rotavirus G-like	Yes	partial VP1	1474	YP_008136242.1 VP1 [Rotavirus G chicken/03V0567/DEU/2003]	62	3.20E-21
Austrlalian Shelduck	Picornaviridae	Unassigned	Australian Shelduck Sapelovirus-like	Yes	No	360	YP_164335.1 polyprotein [Avian sapelovirus]	47.8	0
Austrlalian Shelduck	Coronaviridae	Gammacoronavirus	Gammacoronavirus	No	Yes	13176	AKF17722.1 ORF1ab polyprotein [Duck coronavirus]	83.2	0
Austrlalian Shelduck	Parvoviridae	Parvovirus	Australian Shelduck Parvovirus	Yes	No	341	ANY57893.1 non-structural protein 1 [Rat parvovirus 2]	62.5	1.20E-40

Wood Duck	Hepandaviridae	Avidepadnavirus	Wood Duck Picornavirus	Yes	No	389	ADP55745.1 large S protein [Duck hepatitis B virus]	88.2	1.60E-173
Pink-eared Duck	Astroviridae	Avastrovirus	Pink-eared Duck Astrovirus Group 2	Yes	No	87	AID55205.1 non-structural polyprotein [Duck astrovirus CPH]	61.8	2.80E-44
Pink-eared Duck	Caliciviridae	Unassigned	Pink-eared Duck Calicivirus A	Yes	Yes	6592	YP_009028574.1 polyprotein [Goose calicivirus]	67.3	0
Pink-eared Duck	Caliciviridae	Unassigned	Pink-eared Duck Calicivirus B	Yes	No	747	YP_009028574.1 polyprotein [Goose calicivirus]	45.6	3.30E-154
Pink-eared Duck	Picornaviridae	Megrivirus	Pink-eared Duck Megrivirus	yes	Yes	3658	YP_009030047.1 polyprotein [Duck megrivirus]	68	0
Pink-eared Duck	Picobirnaviridae	Picobirnavirus	Pink-eared Duck Picobirnavirus	Yes	Yes	1742	AGK45545.1 RNA-dependent RNA polymerase; partial [Fox picobirnavirus]	78	3.40E-264
Pink-eared Duck	Picornaviridae	Sapelovirus	Pink-eared Duck Picornavirus	Yes	Yes	2181	YP_164335.1 polyprotein [Avian sapelovirus]	36.9	0
Pink-eared Duck	Reoviridae	Rotavirus	Duck Rotavirus G-like	Yes	partial VP1	2791	AGW95854.1 VP1 [Rotavirus G pigeon/HK18]	65.1	5.90E-61
Pink-eared Duck	Orthomyxoviridae	Influenza A	Influenza A	H3N1	mostly	536	ADU17311.1 hemagglutinin [Influenza A virus (A/northern pintail/Interior Alaska/8BM3582/2008(H3N8))]	97.7	1.40E-42

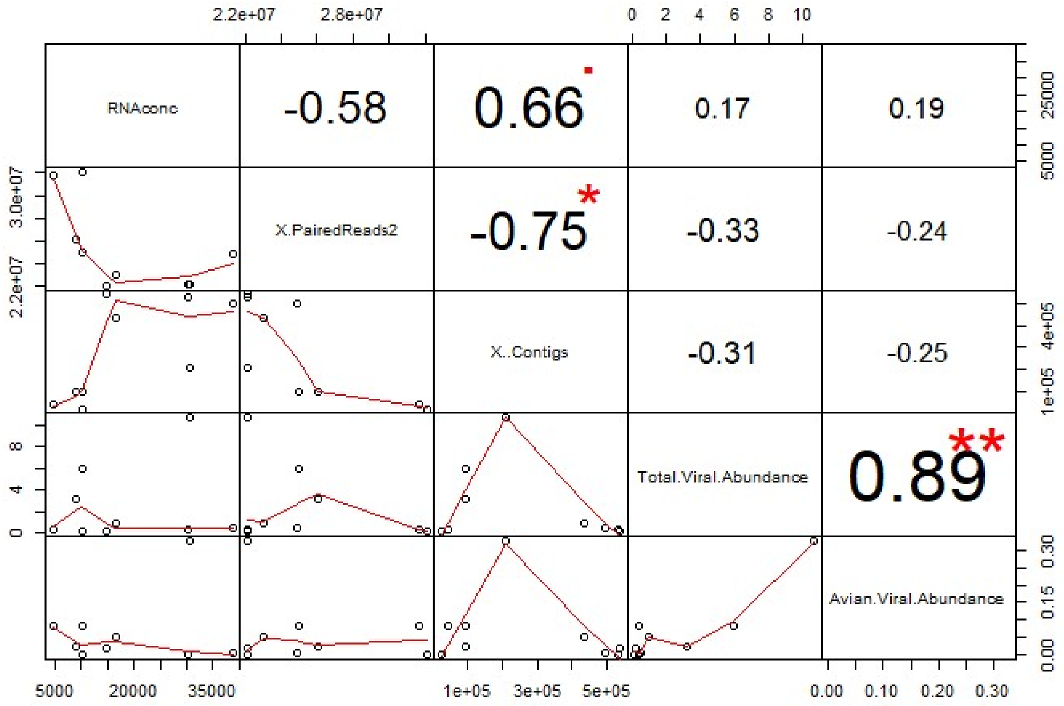

Figure S1. Correlation of laboratory and sequencing metrics. Correlation between pooled sample RNA concentration, number of paired reads generated, number of contigs, total viral abundance and avian viral abundances. Where there are significant correlations between total viral abundance and avian viral abundance, and the number of paired reads and number of contigs generated, there is no statistically significant correlation (presented as asterisks) between starting RNA concentration and any sequencing metric. Metrics used are presented in Table S1.

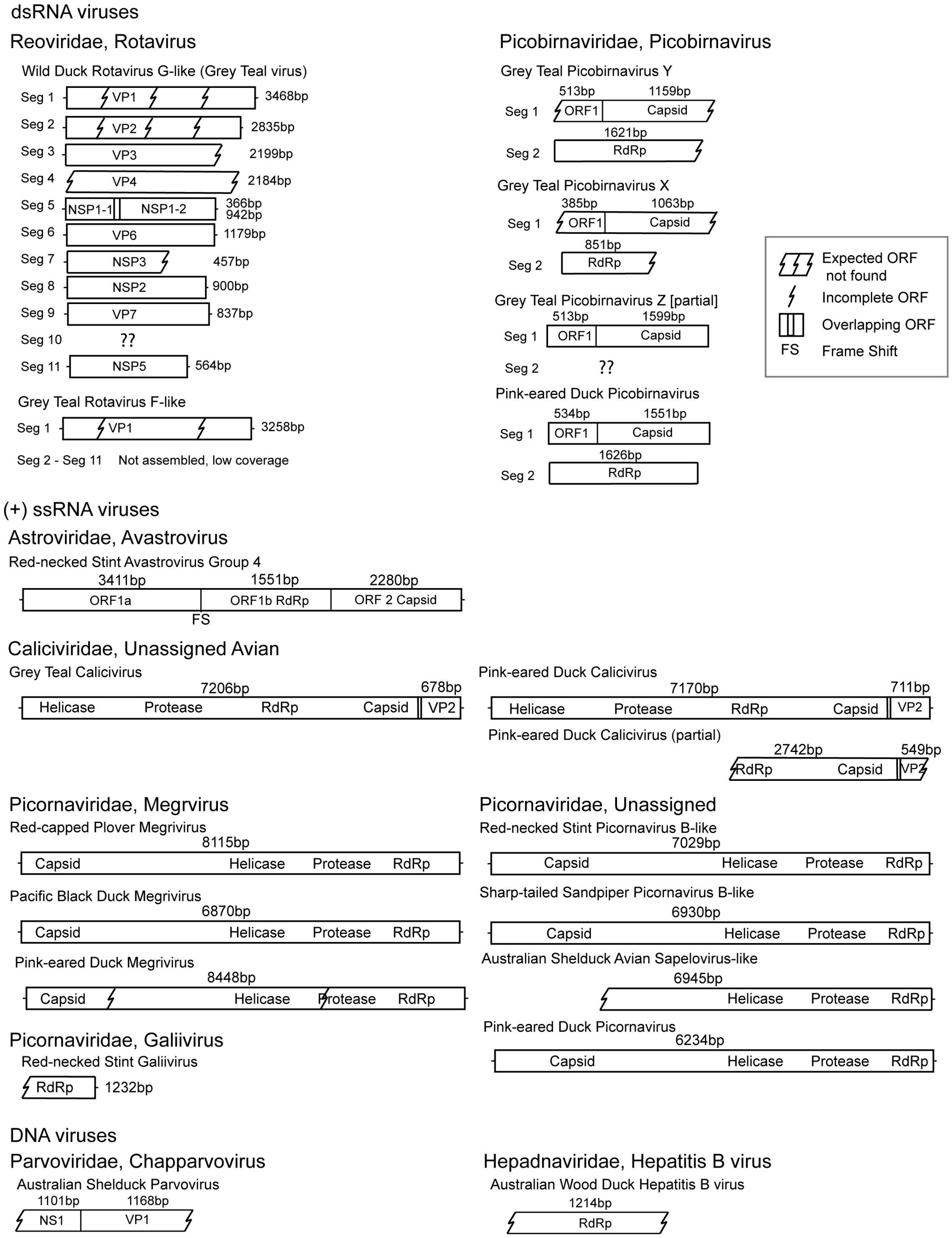

Figure S2. Schematic overview of novel viruses revealed in this study.

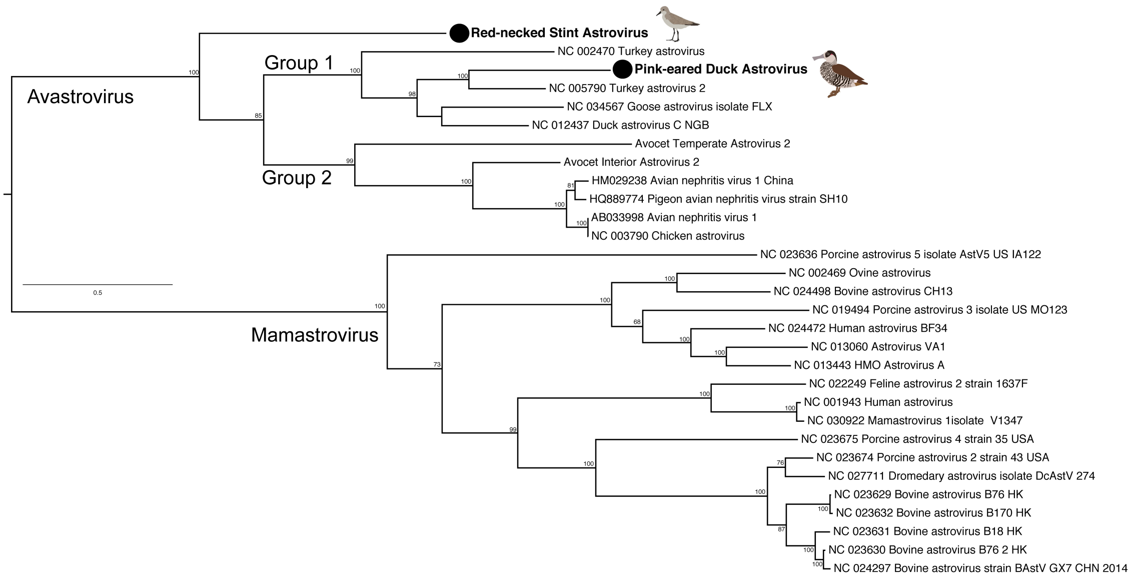

Figure S3. Phylogenetic tree of the ORF1ab, including the RdRp, of Avastroviruses. Viruses identified in this study are denoted with a filled circle and in bold. Pink-eared Duck Astrovirus is a Group 2 virus, Red-necked Stint Astrovirus appears to be a new Group, intermediate between Group 1-2 and Group 3 (as in Figure S4). Group 3 is not shown as there are no full genomes available, but is presented in Figure S4. The tree is mid-point rooted for clarity only. Bootstrap values >70% are shown for key nodes. The scale bar represents the number of amino acid substitutions per site.

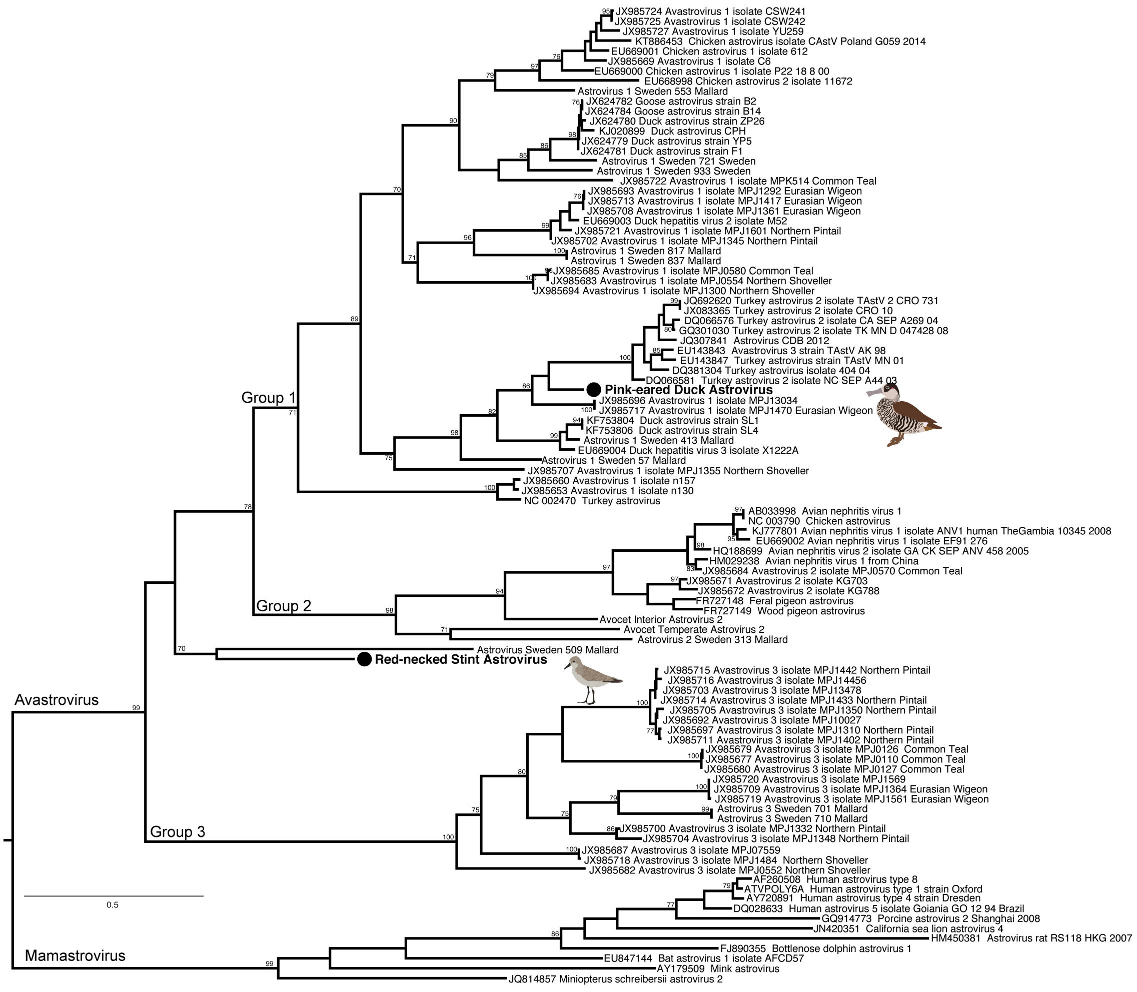

Figure S4. Partial RdRp phylogeny of members of the Avastroviruses. Red-necked Stint Astrovirus, a sister-group to an astrovirus identified in a Swedish Mallard potentially represent a new Group of Astroviruses. The tree is rooted between the avian and mammalian astroviruses. The scale bar indicates the number of nucleotide substitutions per site. Viruses described in this study are marked in bold, adjacent to a filled circle. Bootstrap values >70% are shown for key nodes. The scale bar indicates the number of amino acid substitutions per site. The phylogeny of the full length polyprotein is presented in Figure S2.

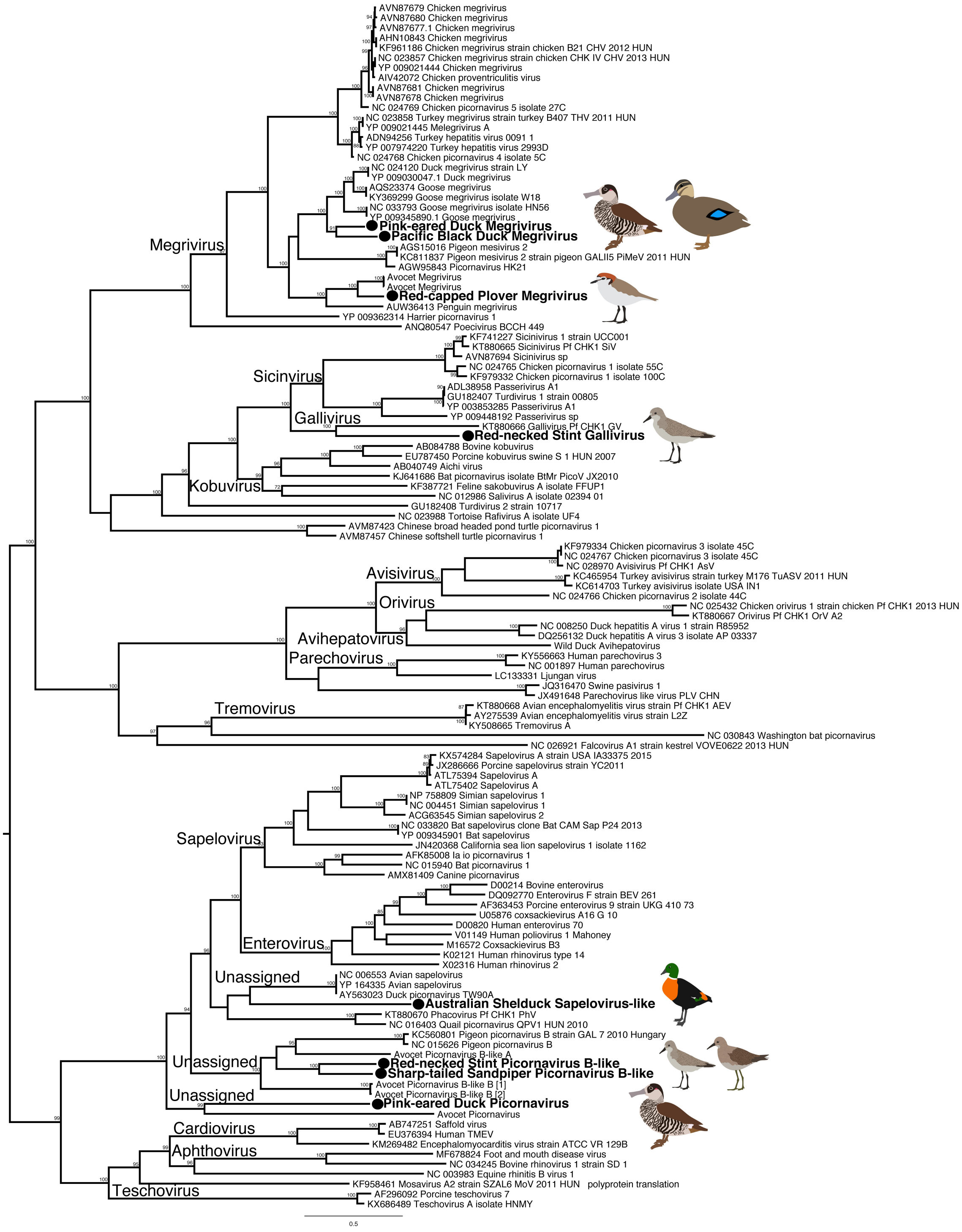

Figure S5. Phylogeny of the polyprotein, containing the RdRp, of select members of the *Picornaviridae*. Reference viruses are those from Boros et al. (2016). The tree was midpoint rooted for clarity only. Viruses described in this study are marked in bold, adjacent to a filled circle. Bootstrap values >70% are shown for key nodes. The scale bar indicates the number of amino acid substitutions per site.

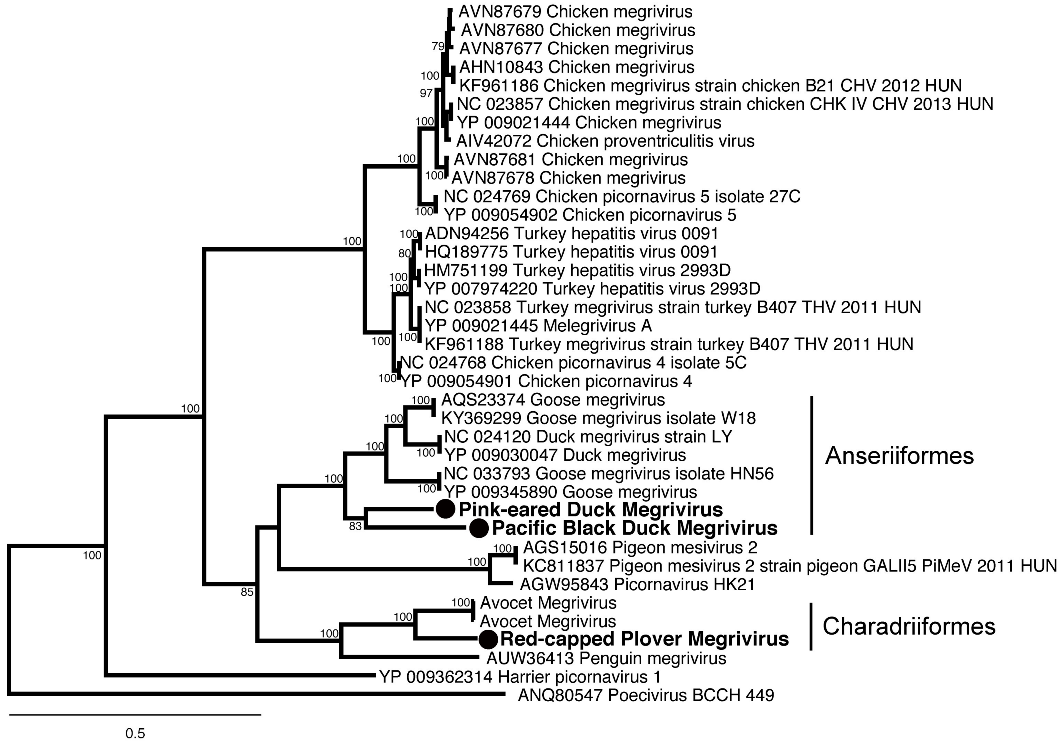

Figure S6. Phylogeny of the polyprotein, containing the RdRp, of select members of the Megriviruses. Poevivirus was set as the outgroup. Viruses described in this study are marked in bold, adjacent to a filled circle. Bootstrap values >70% are shown for key nodes. The scale bar indicates the number of amino acid substitutions per site. Broadly, there is a trend for viral species isolated from Chickens, Turkeys, Pigeons, Anseriifromes and Charadriiformes to form independent, monophyletic groups.

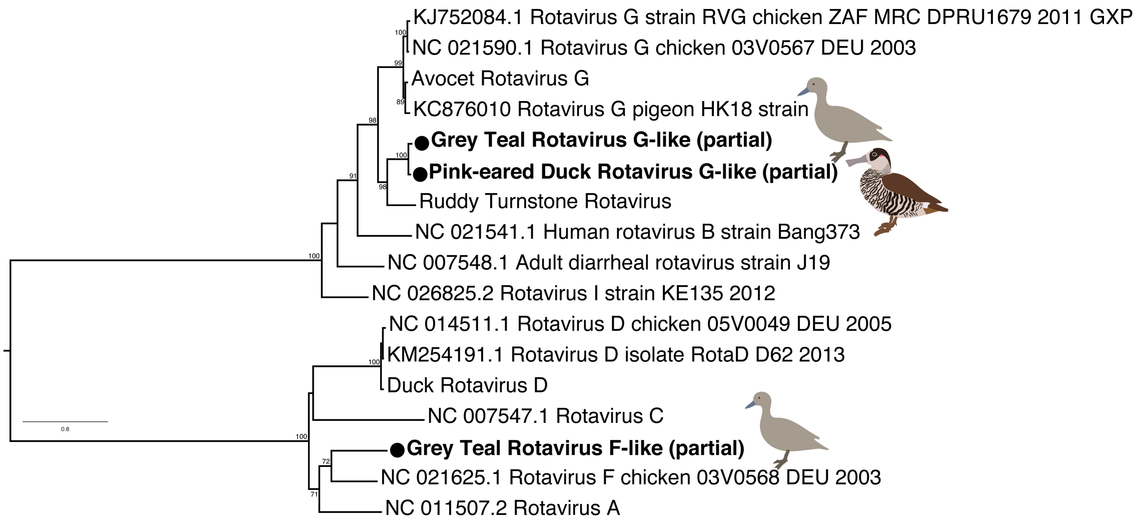

Figure S7. Phylogenetic tree of the VP1, containing the RdRp, of the rotaviruses. The sequences generated in this study are indicated by a filled circle and are in bold. The tree is midpoint rooted for clarity only. Bootstrap values >70% are shown for key nodes. The scale bar indicates the number of amino acid substitutions per site

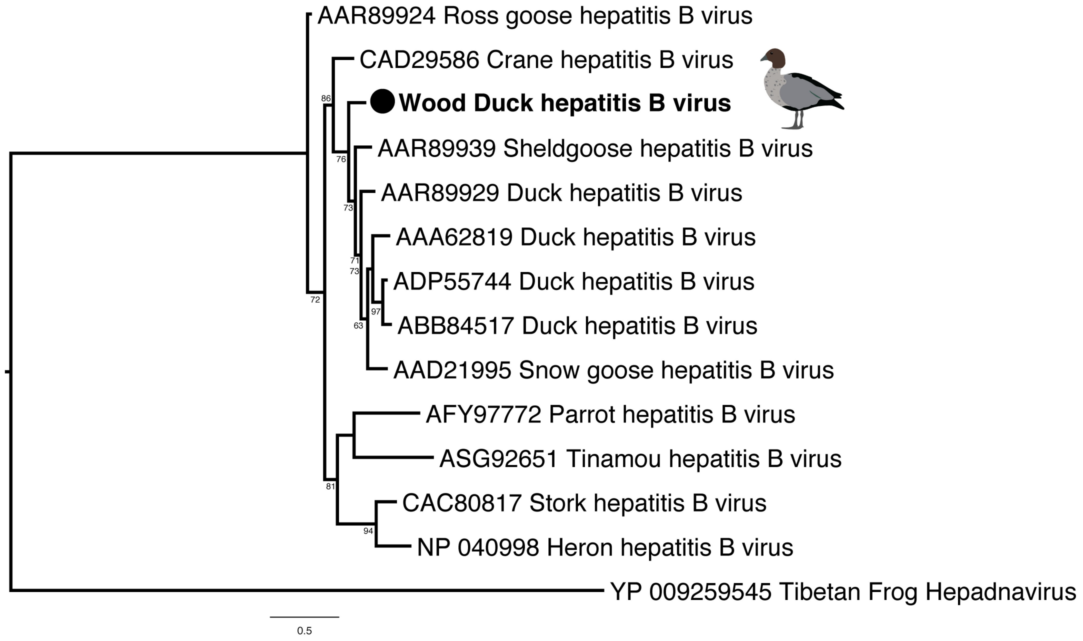

Figure S8. Phylogenetic tree of the P protein of avian hepatitis B viruses. The sequence generated in this study are indicated by a filled circle and are in bold. Tibetan Frog Hepadnavirus is set as the outgroup. Bootstrap values >70% are shown for key nodes. The scale bar indicates the number of amino acid substitutions per site

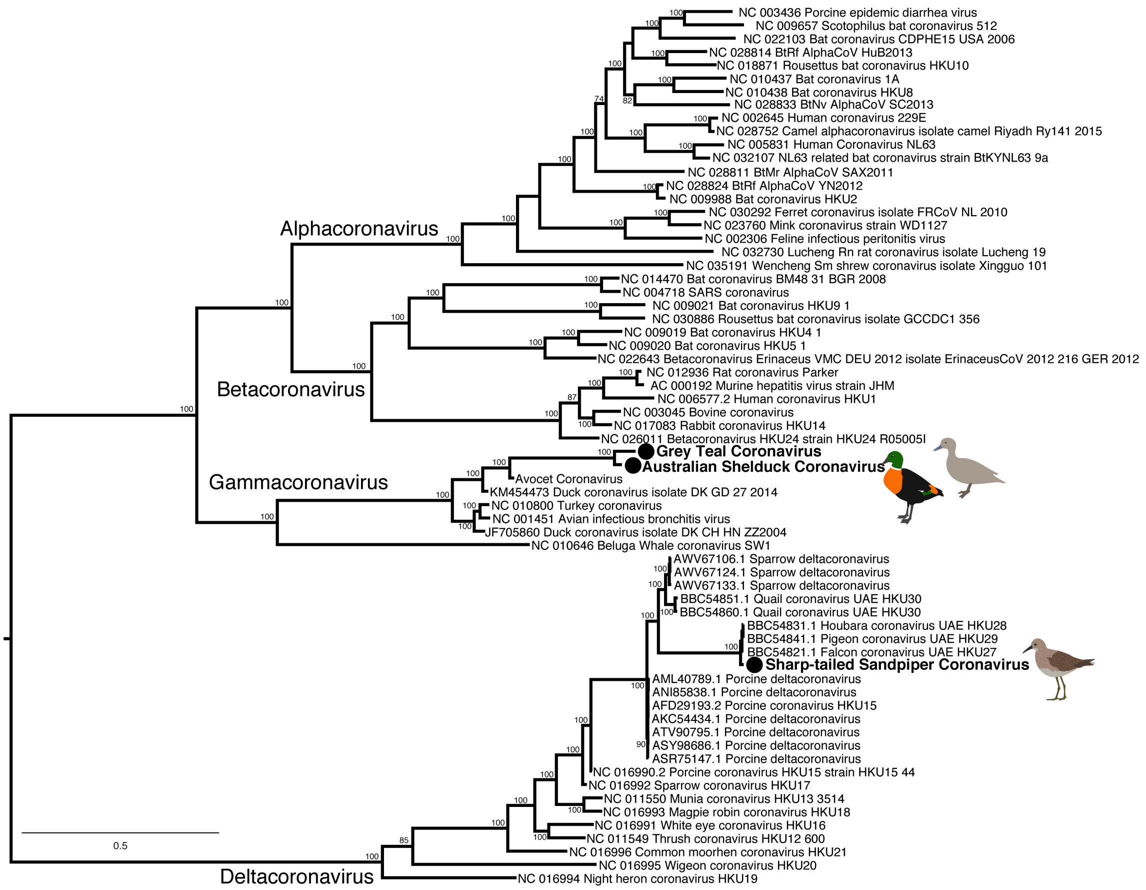

Figure S9. Phylogenetic tree of the ORF1ab, containing the RdRp, of the *Coronaviridae*. The sequences generated in this study are indicated by a filled circle and are in bold. The tree is midpoint rooted for clarity only. Bootstrap values >70% are shown for key nodes. The scale bar indicates the number of amino acid substitutions per site. Partial gammacoronavirus and deltacoronavirus phylogenies, allowing for the inclusion of numerous wild bird sequences, are presented in Figure S10 and S11, respectively.

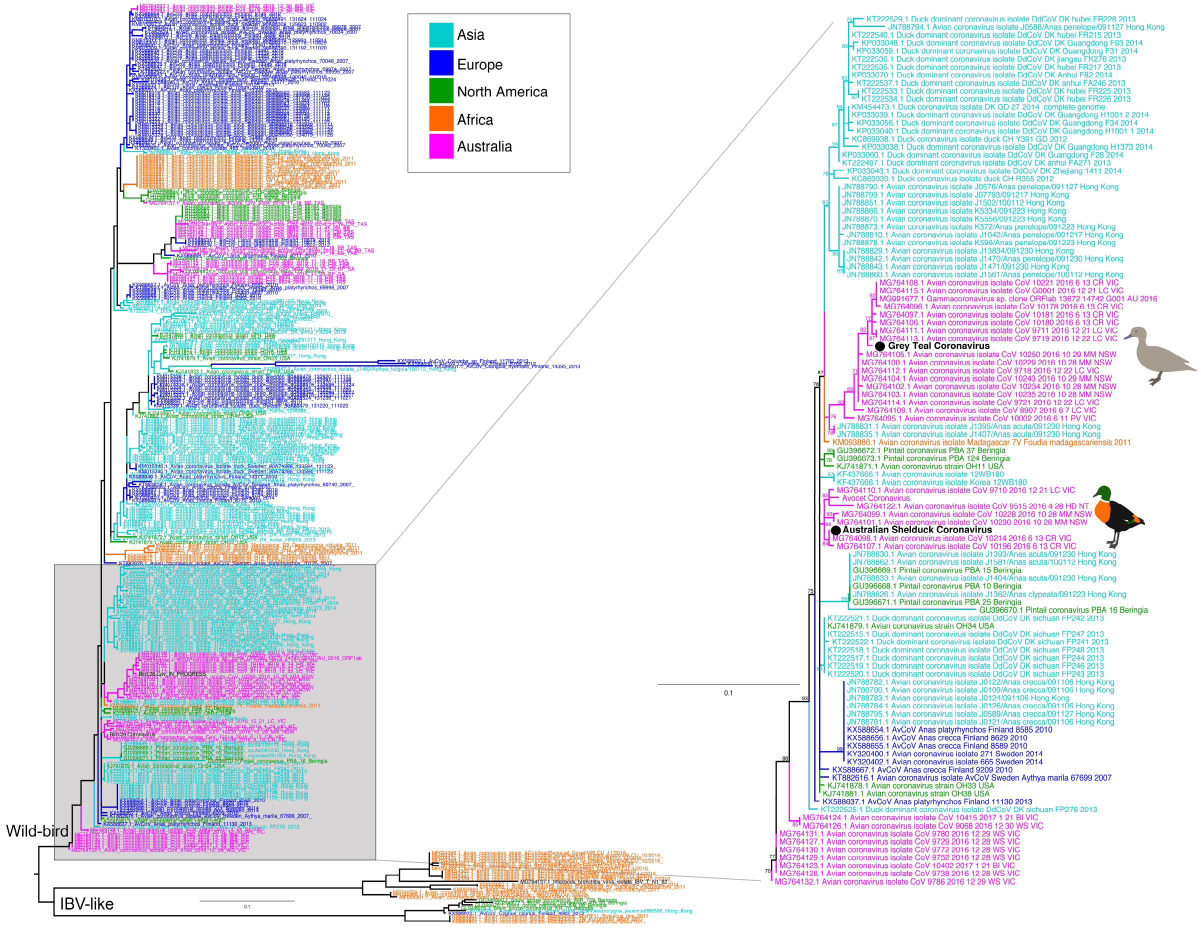

Figure S10. Phylogeny of the partial RdRp of gammacoronaviruses. The tree shows all current wild bird sequences, the majority of which form a clade (“Wild-bird”) that is a sister-group to the Infectious Bronchitis virus (IBV) and Infectious-bronchitis virus-like viruses. The clade comprising IBV and IBV-like viruses was set as the outgroup. Tips are coloured by geographic origin. The subsection of the tree within the grey box has been expanded to allow for legibility of tip names, and the viruses identified in this study are in bold and denoted by a filled circle. Bootstrap values >70% are shown for key nodes .The scale bar represents the number of nucleotide substitutions per site.

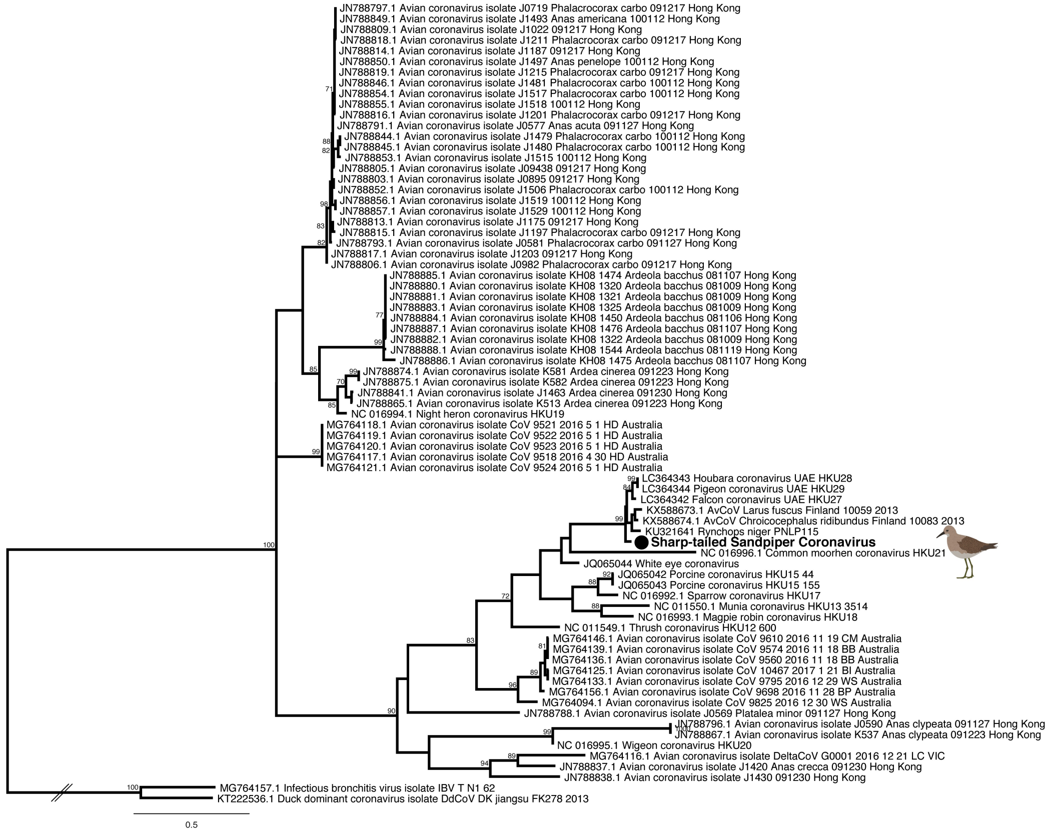

Figure S11. Phylogeny of the partial RdRp of deltacoronaviruses. Two representative gammacoronaviruses are set as the outgroup. Viruses identified in this study are in bold and denoted by a filled circle. Bootstrap values >70% are shown for key nodes. The scale bare represents the number of nucleotide substitutions per site.

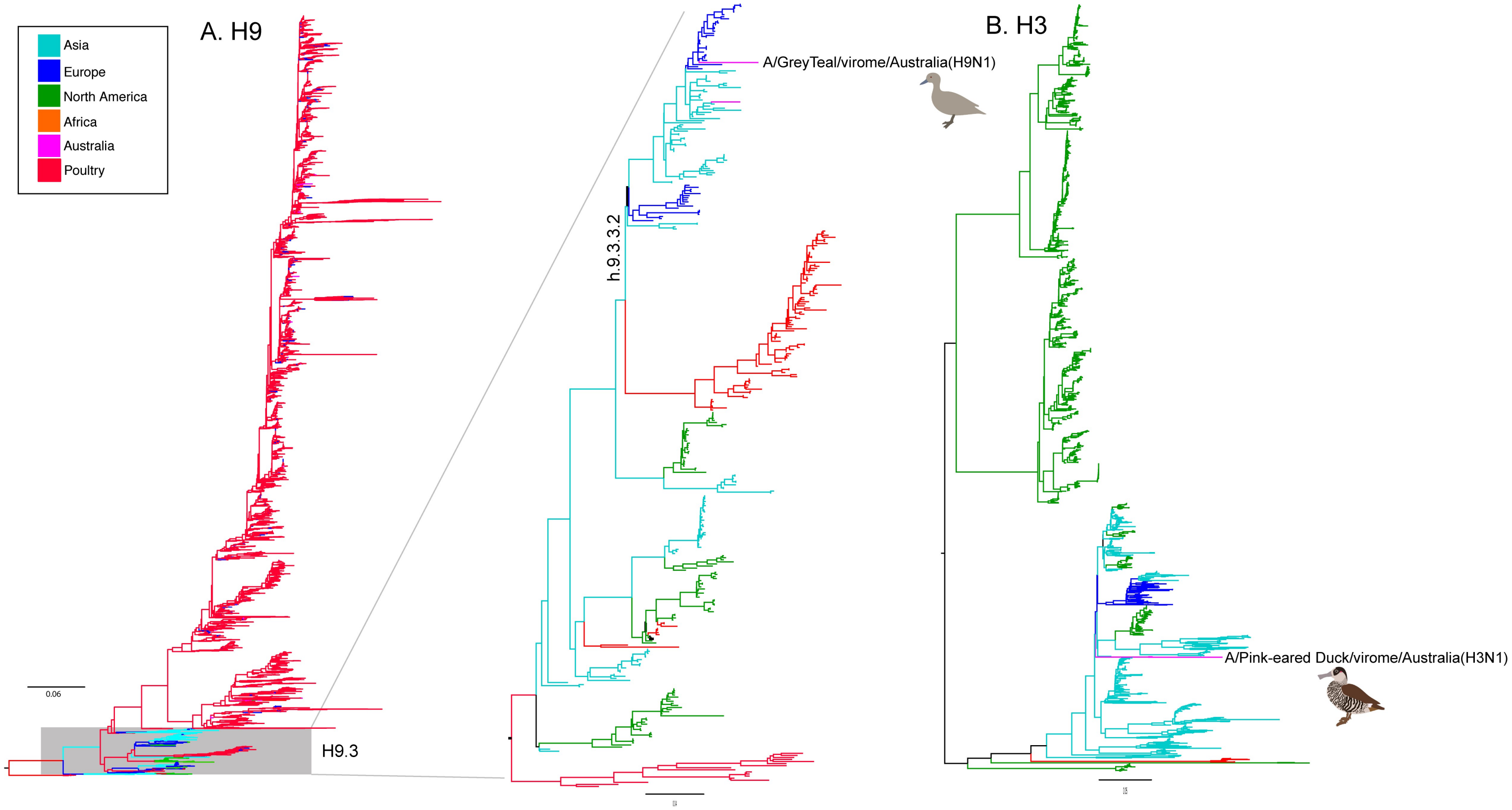

Figure S12. Phylogenies of the HA genes of H9 and H3 influenza A viruses containing all wild bird sequences. (A) The H9.3 clade is identified by a grey box on the global H9 phylogeny, in which clade H9.1 is set as the outgroup. Clade H9.3 has been expanded, and is rooted by more distantly related H9 sequences; subclade H9.3.3.2 is further identified. Representatives viruses in this clade are those identified by Jiang et al. (2012). (B) The H3 phylogeny was mid-point rooted, corresponding to a major biogeographic division between Eurasia and the Americas. The phylogenetic position of the viruses sequenced in this study by their designations: A/Grey Teal/virome/Australia/2016(H9N1) and A/Pink-eared Duck/virome/Australia/2016(H3N1), respectively. Bootstrap values >70% are shown for key nodes The scale bare indicates number of nucleotide substitutions per site.

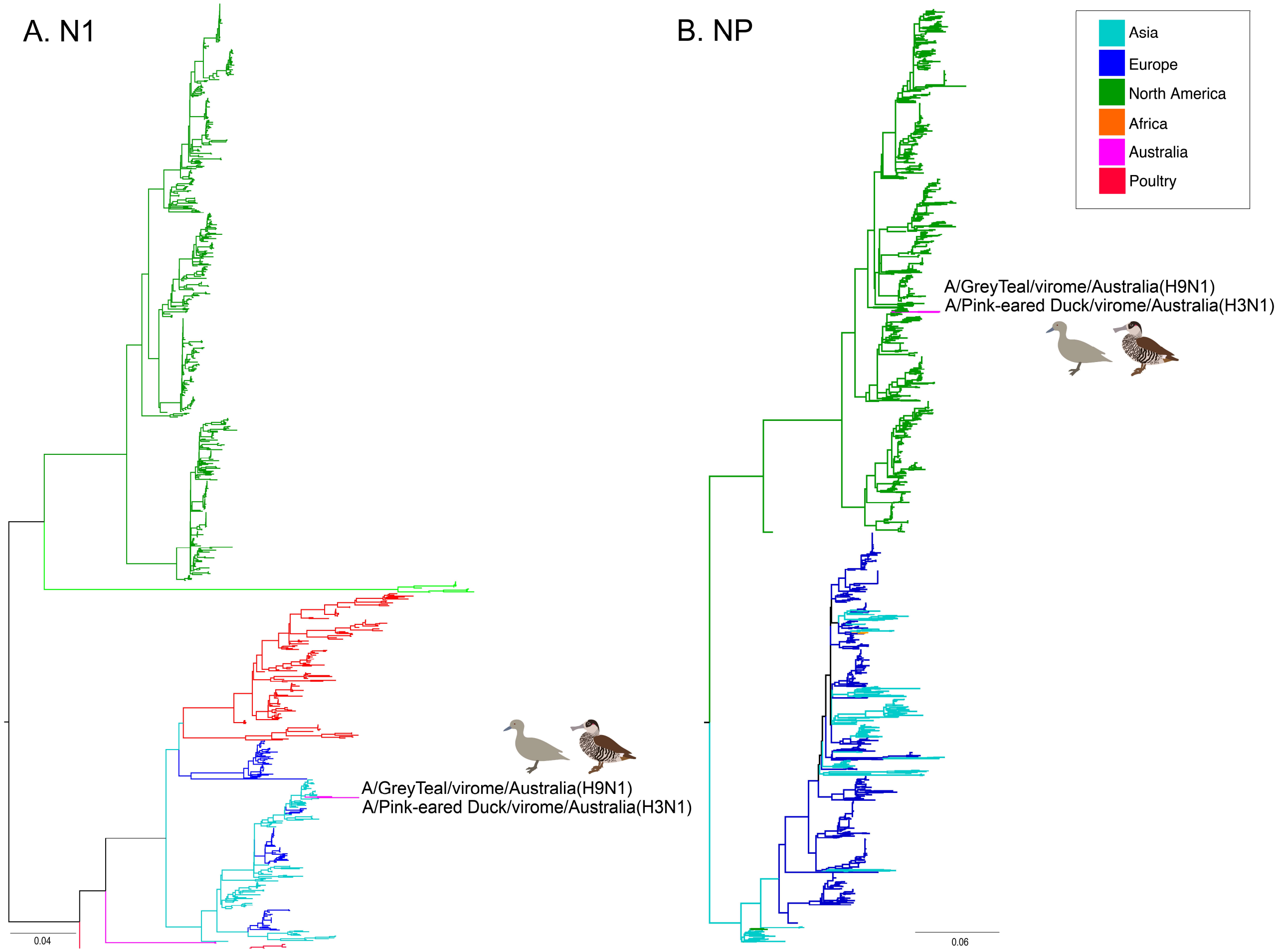

Figure S13. Phylogenies of the NA and NP genes of influenza A viruses, containing all wild bird sequences. Trees were midpoint rooted, corresponding to a major biogeographic division between Eurasia and the Americas. The phylogenetic position of the viruses sequenced in this study by their designations: A/Grey Teal/virome/Australia/2016(H9N1) and A/Pink-eared Duck/virome/Australia/2016(H3N1) respectively. Bootstrap values >70% are shown for key nodes. The scale bar indicates number of nucleotide substitutions per site

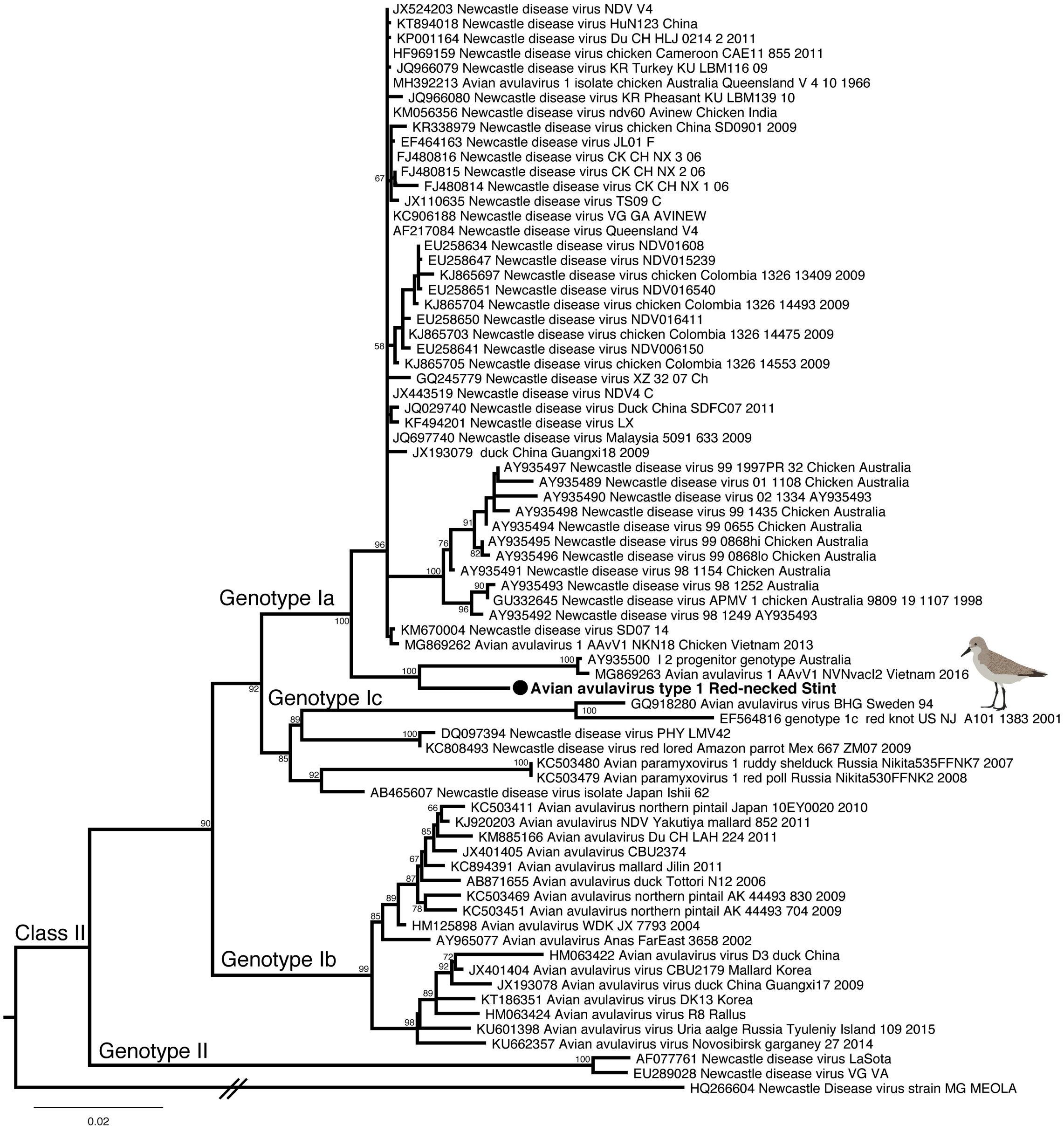

Figure S14. Phylogeny of the F gene of avian avulavirus Type 1 Class II Genotype 1. A Class I virus, strain MG MEOLA was set as the outgroup. The virus identified in this study is in bold and denoted by a filled circle in Genotype 1a, with a low pathogenic cleavage site: GRQGR*L. Bootstrap values >70% are shown for key nodes. The scale bar represents the number of nucleotide substitutions per site.

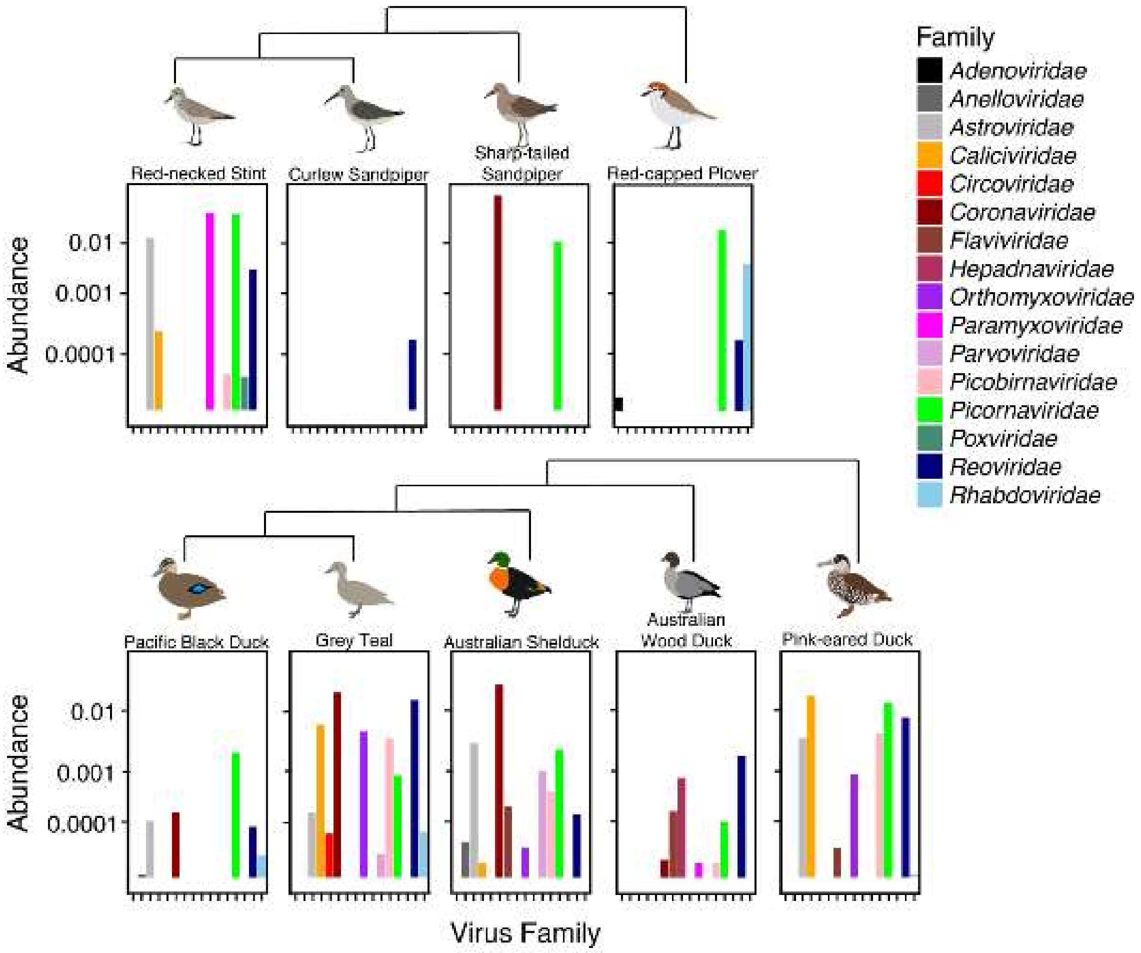

Figure S15. Abundance of avian viral families identified in each library. Libraries are arranged taxonomically, with cladograms illustrating species relationships within the Charadriiformes and Anseriiformes. Taxonomic identification presented is that of the top blastx hit of all avian viral contigs. Alpha diversity metrics are presented in Figure S16.

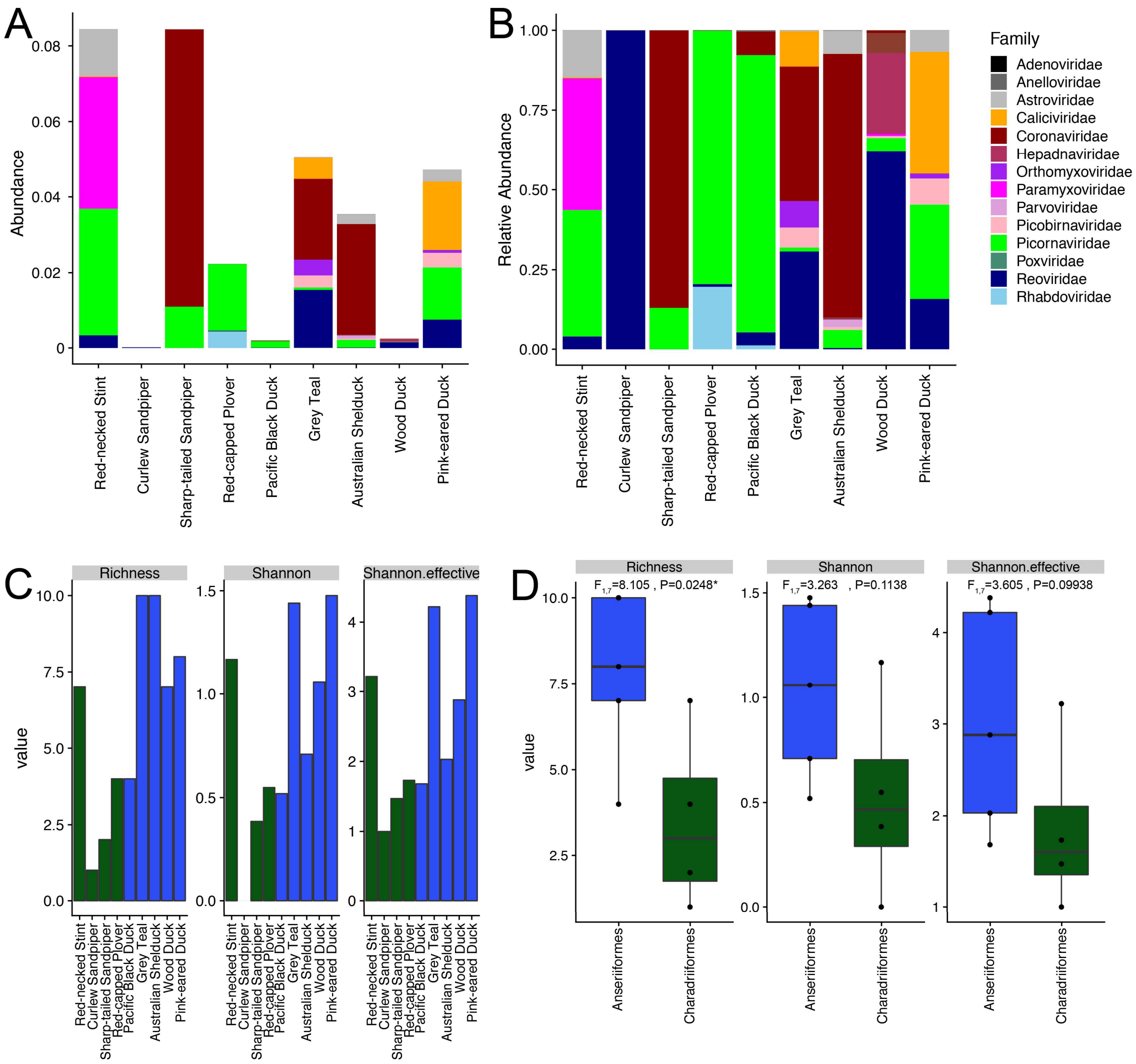

Figure S16. Alpha diversity of avian viral families identified in each library. (A) Abundance of viral families in each library and (B) relative abundance of viral families in each library. (C) Alpha diversity metrics calculated for each library and (D) for avian order. Differences in alpha diversity between Anseriiformes and Charadriiformes were calculated using a linear model following a box-cox transformation. In panels C and D samples from Charadriiformes are in green and from Anseriiformes in blue. Panel A is reproduced in Fig 1.

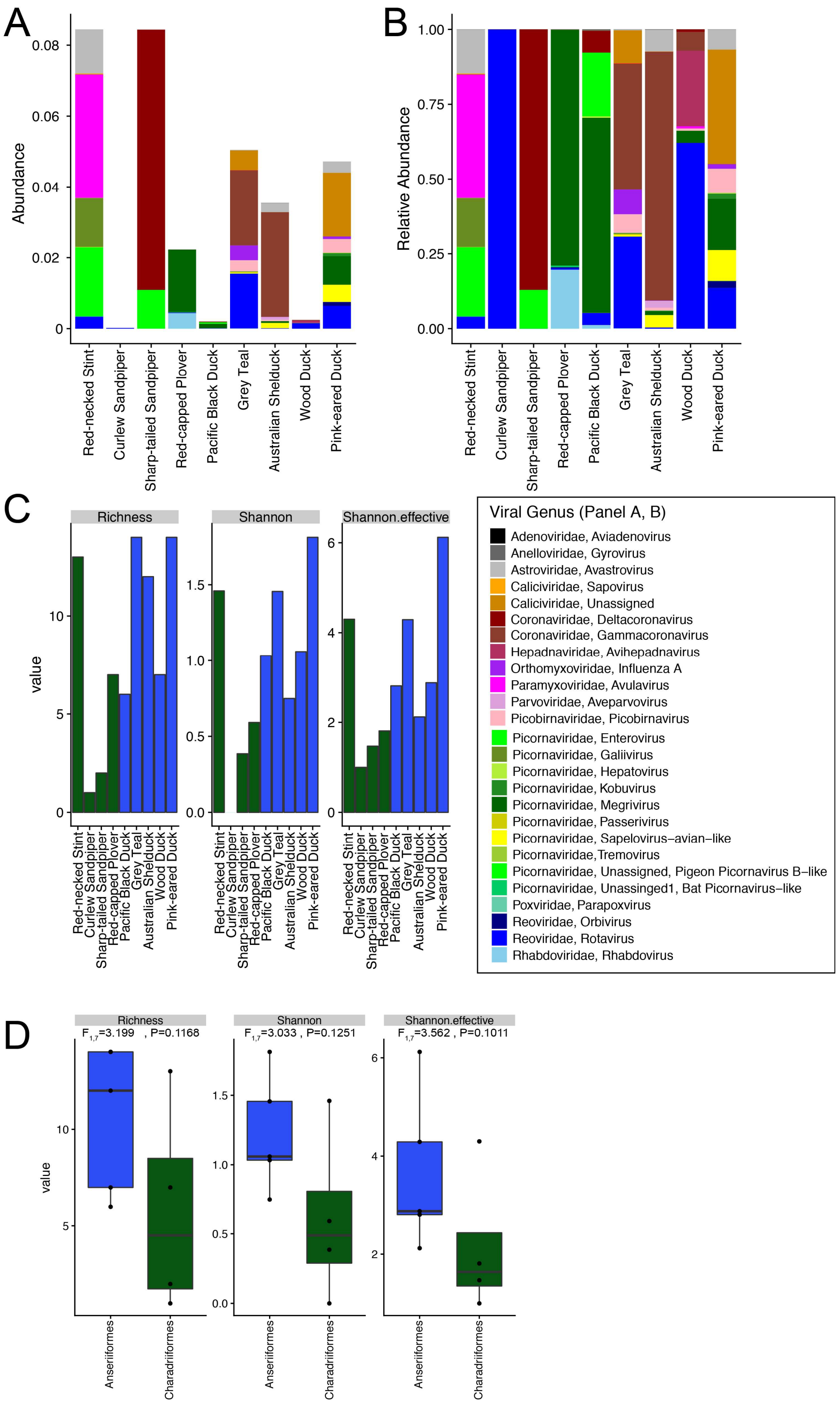

Figure S17. Alpha diversity of avian viral genera identified in each library. (A) Abundance of viral genera in each library and (B) relative abundance of viral genera in each library. (C) Alpha diversity metrics calculated for each library and (D) for avian order. Differences in alpha diversity between Anseriiformes and Charadriiformes were calculated using a linear model following a box-cox transformation. In panels C and D samples from Charadriiformes are in green and from Anseriiformes in blue.

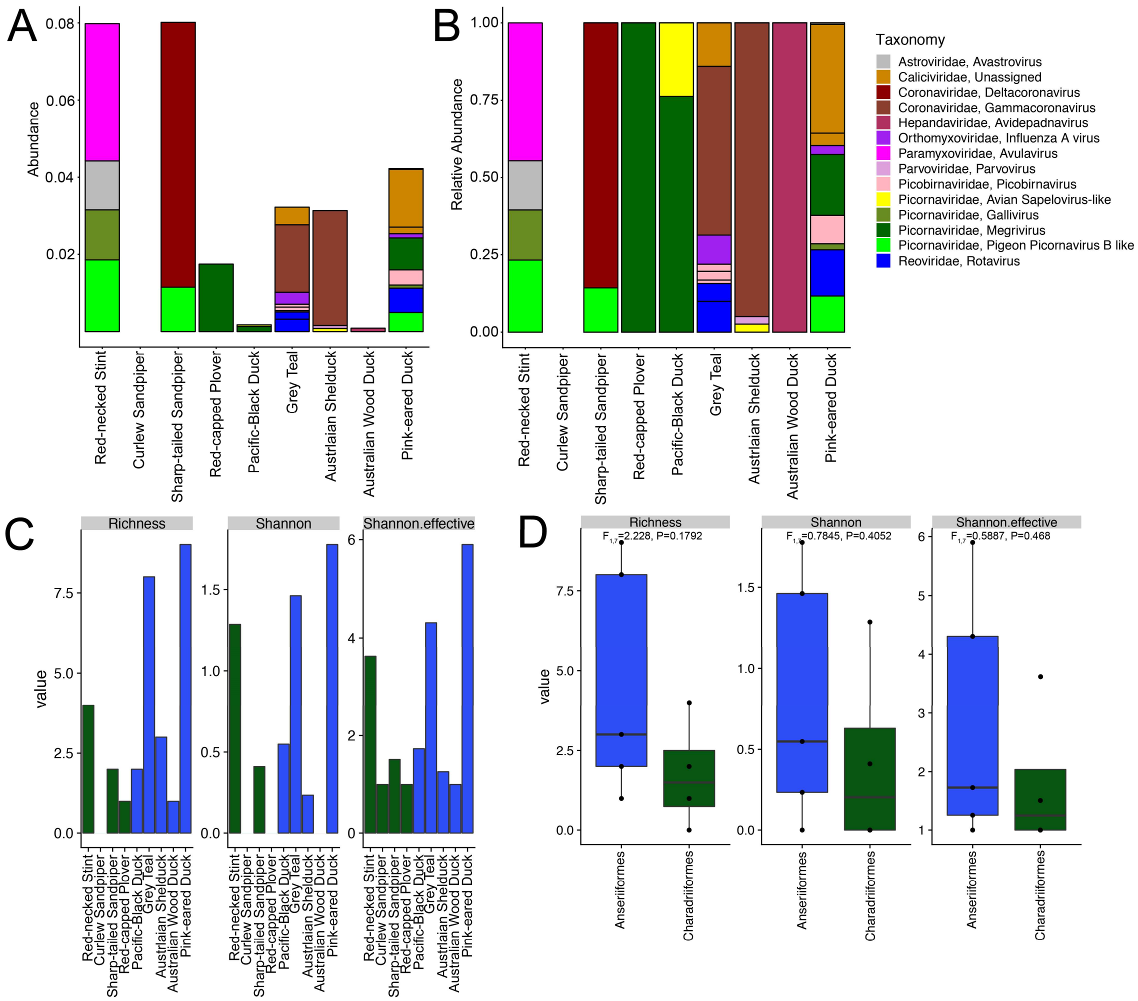

Figure S18. Alpha diversity of avian viral species characterised in this study. (A) Abundance of viral genera in each library and (B) relative abundance of viral genera in each library. (C) Alpha diversity metrics calculated for each library and (D) for avian order. Differences in alpha diversity between Anseriiformes and Charadriiformes were calculated using a linear model following a box-cox transformation. In panels C and D samples from Charadriiformes are in green and from Anseriiformes in blue. Viral species and abundances are presented in Table S3.

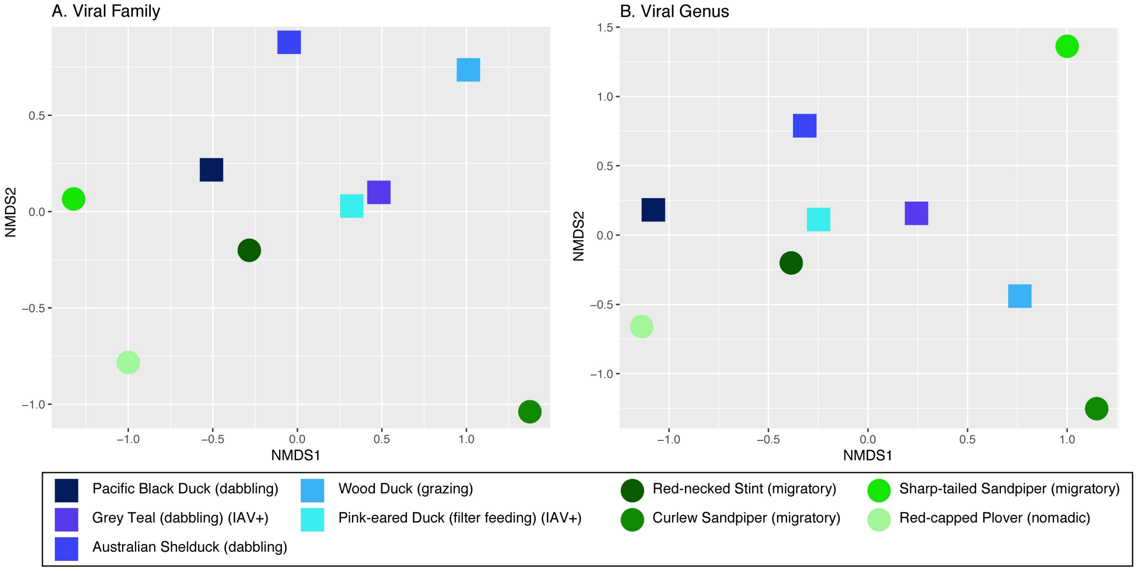

Figure S19. Non-metric multidimensional scaling (NMDS) plot applying the bray curtis dissimilarity matrix for viral abundance and diversity. Panel A uses the viral level library composition and B uses viral genera level library composition. Anseriiform libraries are in shades of blue, and charadriiformes in shades of green.

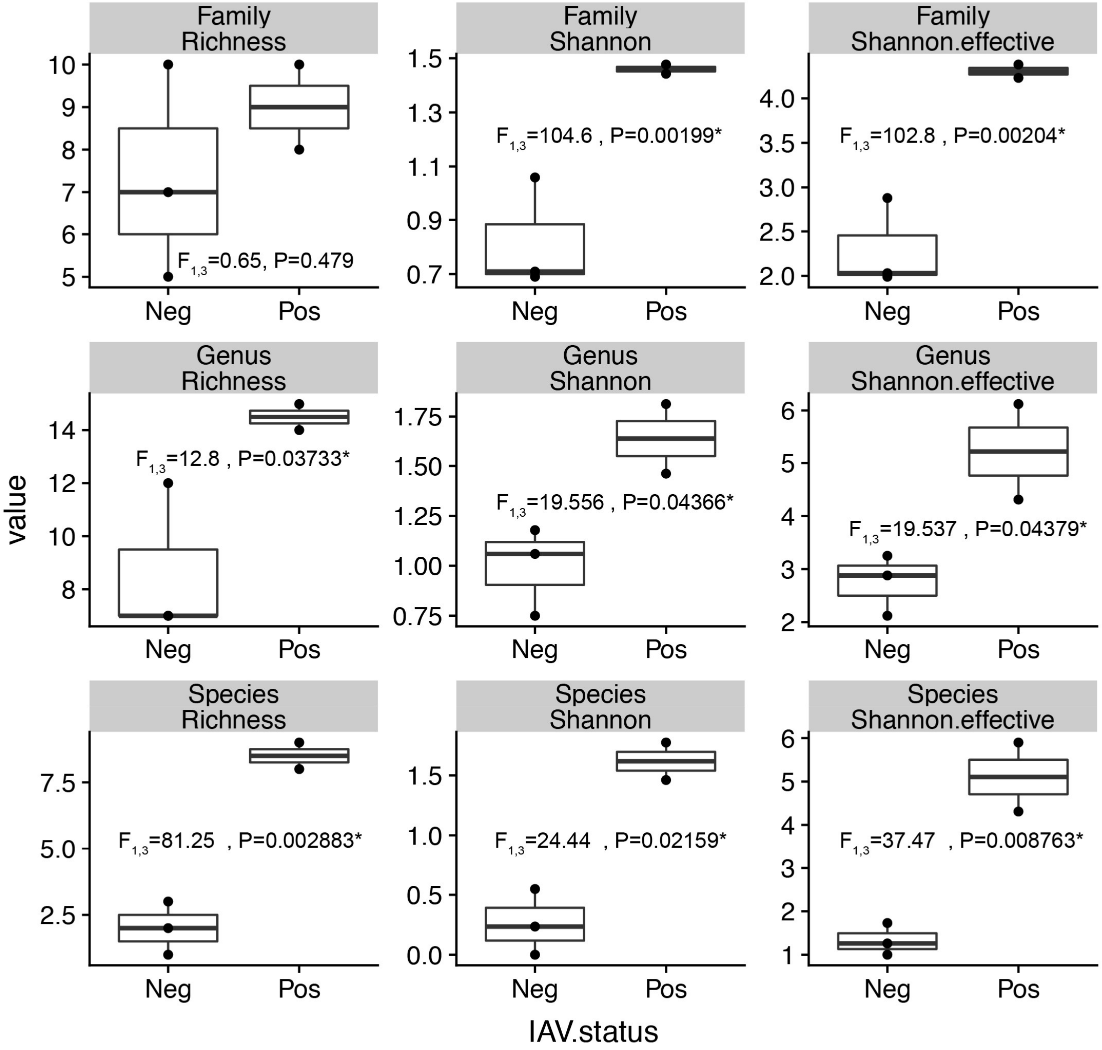

Figure S20. Effect of IAV on Alpha Diversity of Anseriiform viromes. There is a clear trend whereby the median and inter quartile range of alpha diversity is higher in Anseriiform libraries that are positive for influenza A virus as compared to those that are negative. The difference in alpha diversity given IAV status was tested using a linear model following a box-cox transformation.

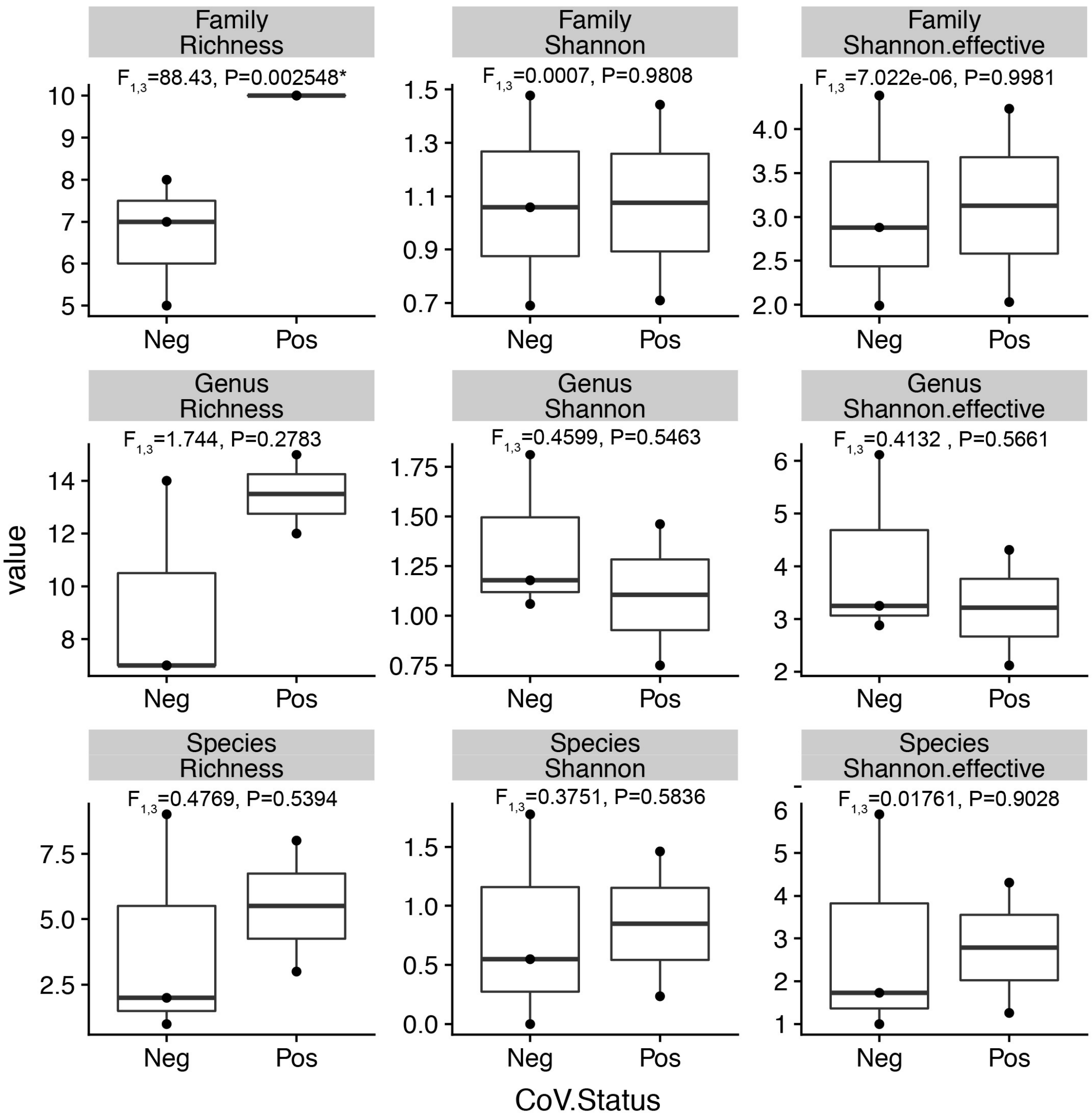

Figure S21. Effect of coronavirus on alpha diversity of Anseriiform viromes. The difference in alpha diversity given CoV status was tested using a linear model following a box-cox transformation.

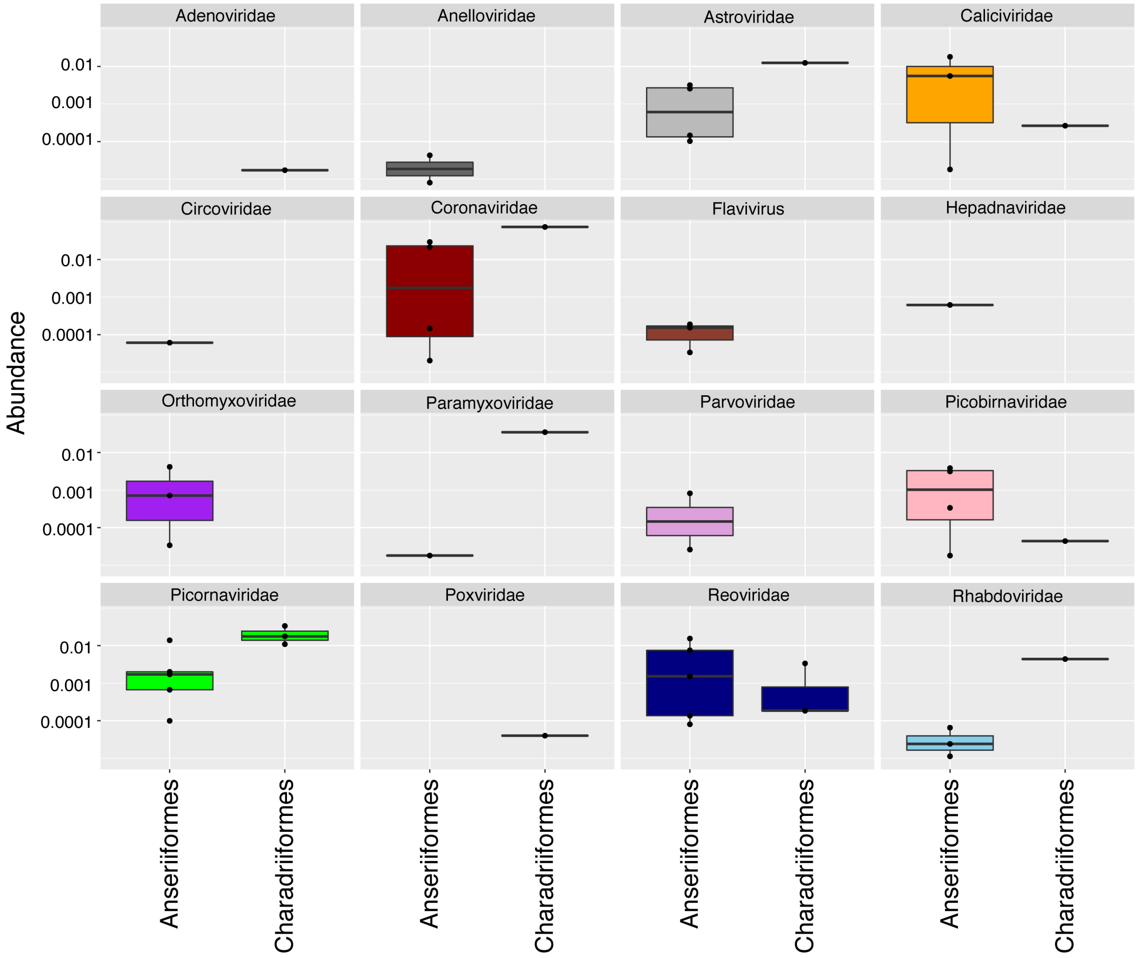

Figure S22. Differential abundance of viral families in libraries from Anseriiformes and Charadriiformes.

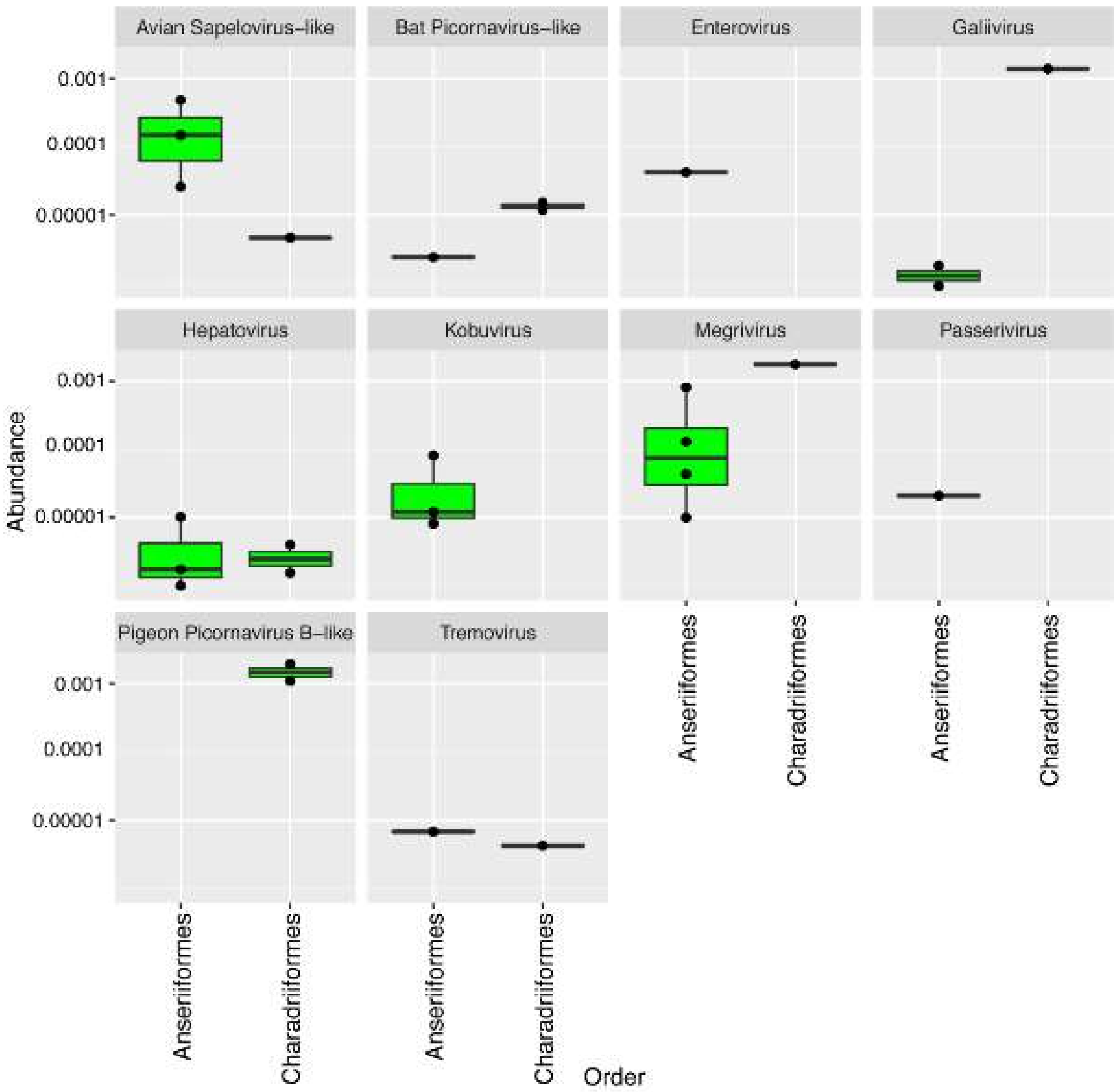

Figure S23. Differential abundance of viral genera in the family *Picornaviridae* in libraries from Anseriiformes and Charadriiformes.

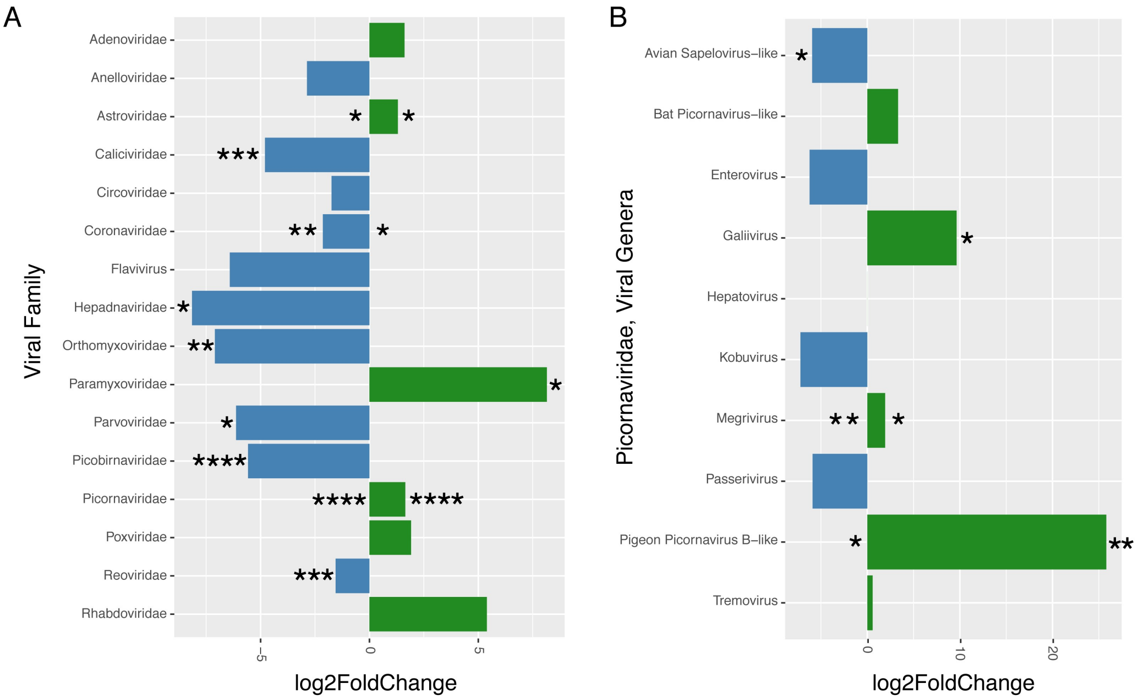

Figure S24. Differential abundance (log 2 fold change) of viruses. (A) Viral families and (B) viral genera in the family *Picornaviridae* in libraries from Anseriiformes and Charadriiformes. Viral families and genera that have a higher abundance in Charadriiformes are coloured in green, and Anseriiformes are coloured in blue. Asterisk indicate the number of characterized viruses from either Anseriiformes or Charadriiformes. Differential abundance is plotted in Figure S12 and S13.

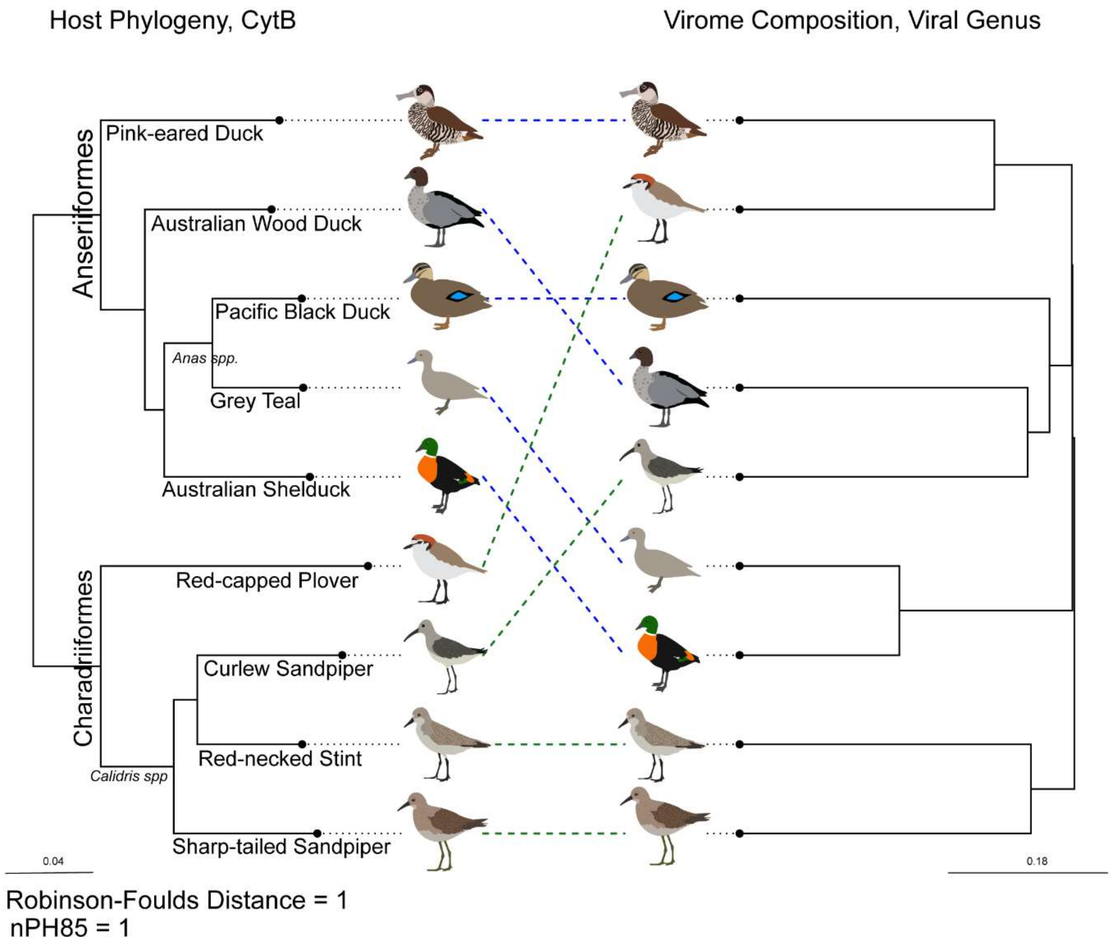

Figure S25. Co-phylogeny demonstrating a discordance between host phylogenetic relationships and virome composition. The host phylogeny was inferred using the cytochrome B gene. The virome composition phylogram was generated by clustering of the Bray-Curtis dissimilarity matrix. Dotted lines between the two phylograms are either green or blue, representing either a Charadriiform or Anseriform species, respectively The relationship was tested using two discordance metrics: Robinson-Foulds and nPH85, where 1 is discordance and 0 is agreement.

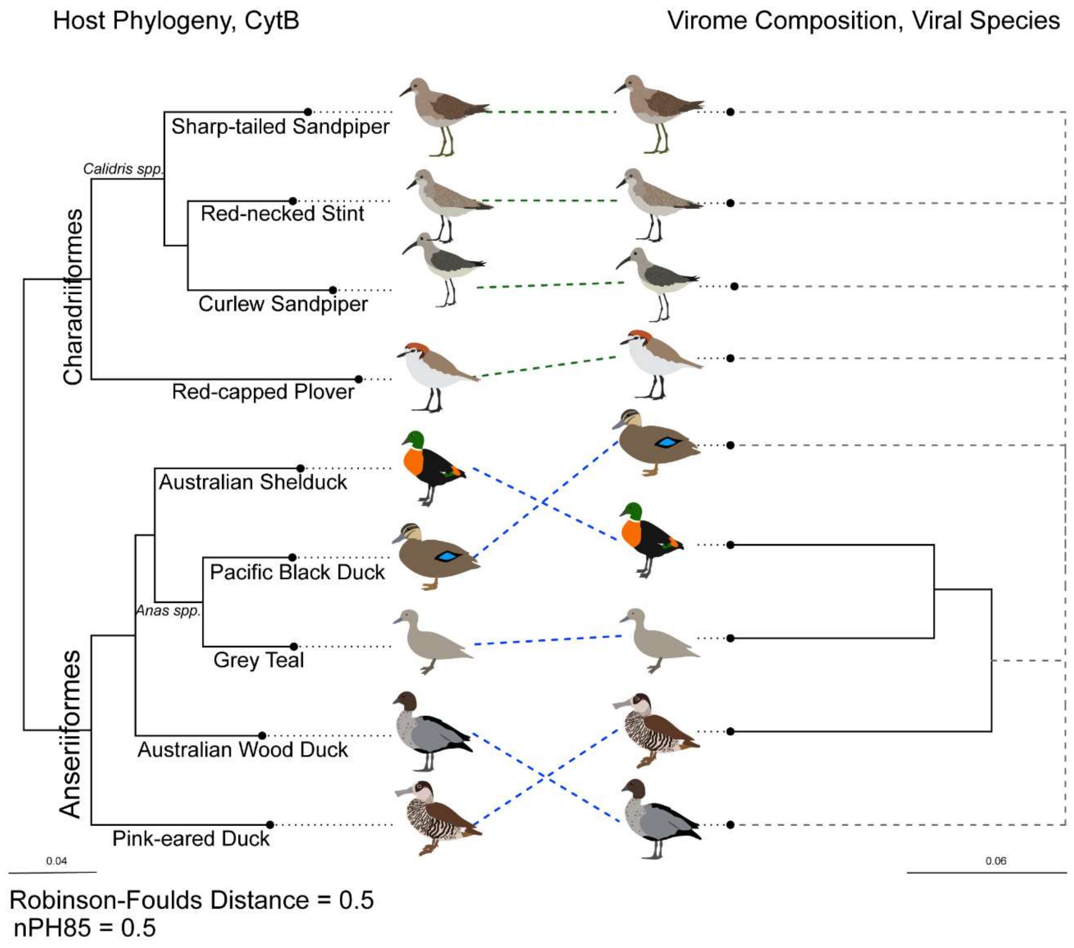

Figure S26. Co-phylogeny demonstrating the discordance between host phylogenetic relationships and virome composition. Host phylogenetic relationships were inferred using the cytochrome B gene. The phylogram of virome composition was generated by clustering of Bray-Curtis dissimilarity matrix. The virome composition phylogram comprises black solid lines between Grey Teal, Pink-eared Duck and Australian Shelduck as viral species were shared between these libraries. As no other libraries shared any viral species, the libraries are connected with grey dashed lines on the phylogram. Dotted lines between the two phylograms are either green or blue, representing either a Charadriiform or Anseriform species, respectively. The relationship was tested using two discordance metrics: Robinson-Foulds and nPH85, where 1 is discordance and 0 is agreement.
